# Potential Role of ROS in Butyrate- and Dietary Fiber-Mediated Growth Inhibition and Modulation of Cell Cycle-, Apoptosis- and Antioxidant-Relevant Proteins in LT97 Colon Adenoma and HT29 Colon Carcinoma Cells

**DOI:** 10.3390/cancers15020440

**Published:** 2023-01-10

**Authors:** Wiebke Schlörmann, Christoph Horlebein, Sabine M. Hübner, Elisa Wittwer, Michael Glei

**Affiliations:** Department of Applied Nutritional Toxicology, Institute of Nutritional Sciences, Friedrich Schiller University Jena, Dornburger Straße 24, 07743 Jena, Germany

**Keywords:** butyrate, β-glucan, colon cancer, chemoprevention, fiber, ROS

## Abstract

**Simple Summary:**

The aim of the present study was to elucidate the potential contribution of oxidative stress to chemopreventive effects of butyrate and fermentation supernatants (FS) of different fiber samples. LT97 and HT29 cells were treated with butyrate or FS with or without *N*-acetyl-cysteine (NAC) and the impact on proliferation and formation of reactive oxygen species (ROS) and expression of cell cycle-, apoptosis-, and antioxidant-relevant proteins was investigated. We were able to demonstrate that butyrate- and FS-mediated chemopreventive effects are modulated differentially in both cells lines by NAC. These results suggest a potential contribution of ROS to the observed chemopreventive effects of butyrate and fiber FS depending on the cell line used. This study provides important insights into the role of ROS in butyrate- and dietary fiber-mediated chemopreventive effects towards colon cancer cells from different transformation stages and it provides a basis for further mechanistic studies.

**Abstract:**

The aim of the present study was to examine whether reactive oxygen species (ROS) contribute to chemopreventive effects of fermentation supernatants (FS) of different dietary fibers (Synergy1^®^, oat-, barley-, yeast β-glucan, Curdlan) and butyrate as a fermentation metabolite. LT97 and HT29 cells were treated with butyrate and FS alone or with *N*-acetyl-cysteine (NAC) and their impact on ROS formation, cell growth, and protein expression (Cyclin D2, p21, PARP, Bid, GPx2) was investigated. Butyrate and FS significantly decreased cell growth. ROS levels were significantly increased, particularly in LT97 cells, while co-treatment with NAC decreased ROS formation and growth inhibitory effects in both cell lines. After treatment with butyrate and FS, Cyclin D2 expression was reduced in LT97 cells and p21 expression was increased in both cell lines. Levels of full-length PARP and Bid were decreased, while levels of cleaved PARP were enhanced. GPx2 expression was significantly reduced by fiber FS in HT29 cells. A notable effect of NAC on butyrate- and FS-modulated protein expression was observed exclusively for PARP and Bid in HT29 cells. From the present results, a contribution of ROS to growth inhibitory and apoptotic effects of butyrate and FS on LT97 and HT29 cells cannot be excluded.

## 1. Introduction

Colorectal cancer represents one of the most common forms of cancer worldwide. Furthermore, it is one of the leading causes of death. With 1.93 million estimated new cases and 0.94 million deaths in 2020, which account for 10% of all cancer cases and for 9.4% of all cancer related deaths, colorectal cancer ranks third regarding incidence and second regarding mortality of all types of cancer [1]. Non-modifiable risk factors such as age and genetic predisposition are related to the development of colorectal cancer. Approximately 35–40% of colorectal cancer cases can be attributed to such hereditary factors resulting in a susceptibility for colon cancer development. Nevertheless, most colon cancer cases are sporadic (60–65%) and induced by sequential genetic mutations and/or epigenetic alterations which are initiated and affected by modifiable risk factors such as lifestyle (e.g., low physical activity, tobacco consumption) and dietary factors (e.g., high intake of red meat, low intake of dietary fiber) [1,2,3,4]. The sequential accumulation of genetic alterations and mutations in tumor-suppressor or proto-oncogenes and the development of colon carcinoma has been described as adenoma-carcinoma sequence by Fearon and Vogelstein [5]. This process can be modulated by so-called “blocking” agents (primary chemoprevention), which inhibit the initiation of colon epithelial cells by enhancing detoxification and reducing toxification (e.g., increase of antioxidant activity, reduction of carcinogen formation, induction of DNA repair) and so-called “suppressing” agents (secondary chemoprevention), which suppress the promotion of colon carcinogenesis by, e.g., inhibiting proliferation and inducing apoptosis of initiated cells [6,7]. The short-chain fatty acid butyrate, a metabolite resulting from dietary fiber fermentation in the colon, mediates such primary and secondary chemopreventive effects, due to its blocking and suppressing activity, respectively. These effects are predominantly mediated by the histone deacetylase inhibitor (HDI) activity of butyrate [6,8,9]. Therefore, the consumption of dietary fiber may contribute to the reduction of colon cancer risk after fermentation and formation of butyrate in the colon. In addition, there is evidence, that a regular intake of fiber particularly from whole grains is associated with a reduced risk for colon cancer [10,11,12,13].

Usually, soluble dietary fiber such as Synergy1^®^ (oligofructose-enriched inulin) and β-glucan from oat or barley are fermented easier than, e.g., insoluble dietary fiber [14,15]. In general, β-glucan from oat and barley is well known for its health-promoting effects regarding cholesterol lowering and glycemic response reducing properties [16,17,18,19]. A recent study also indicated a chemopreventive potential of in vitro digested and fermented β-glucans from oat, barley and other sources (e.g., yeast). Furthermore, a variation in the fermentation profile of the examined β-glucans was observed which is probably due to their different physicochemical properties [20]. The chemopreventive effects of these fibers is thought to be largely mediated by the fermentation metabolite butyrate due to its evidenced function as HDI [6,8,9]. Furthermore, other mechanisms might contribute to chemopreventive effects of butyrate. Studies indicated that butyrate might be involved in the increase of ROS in cancer cells and sensitize such cells towards apoptosis [21,22,23,24]. However, there are only few studies investigating this possible function of butyrate in colon cancer cells [25,26,27]. Therefore, the aim of the present study was to elucidate the potential contribution of ROS to chemopreventive effects of butyrate as a single metabolite as well as of different fermented fibers as complex samples of fermentation metabolites. To consider the physicochemical properties of fibers which may affect the fermentation profile, different β-glucans from various sources and different structures were included in this study. Investigated end points included proliferation, ROS induction, and expression of cell cycle-, apoptosis- and antioxidant-relevant proteins (Cyclin D2, p21, PARP, Bid, GPx2) in colon cancer cells. To consider differences between cells from different transformation grades, two different cell lines were used in the present study. The premalignant LT97 colon adenoma cell line was used as a model of early carcinogenesis [28,29], while HT29 represents cells from an advanced stage of colon carcinogenesis.

## 2. Materials and Methods

### 2.1. Dietary Fibers

For the present study, different types of β-glucans and an oligofructose-enriched inulin (Synergy1^®^) were used. Β-Glucans from oat and barley (β-1,3; β-1,4-glucan; linear), yeast (β-1,3; 1,6-glucan; branched), and *Alcaligenes faecaelis* (Curdlan, β-1,3-glucan; linear) were purchased from Megazyme Ltd. (Bray, Ireland). Synergy1^®^ was supplied by Beneo (Mannheim, Germany).

### 2.2. In Vitro Digestion and Fermentation of Dietary Fiber

In vitro digestion and fermentation experiments were performed as described previously [30] according to a protocol established by Stein et al. [31] who combined the in vitro digestion and fermentation procedures described by Aura et al. [32] and Barry et al. [33], respectively. For these experiments, 0.5 g of each dietary fiber sample were used equivalent to 10 g/L fermentable substance according to Barry et al. [33], while a blank control (batch without fermentable substance) served as fermentation negative control. In brief, samples were resuspended in fermentation buffer (0.1 M KH_2_PO_4_ in aqua bidest., pH 7.0) followed by simulated digestion conditions of mouth, stomach, and small and large intestine as described [30]. All steps of the simulated digestion were carried out at 37 °C in a shaking water bath. Conditions of the large intestine were simulated by mixing pre-digested samples with equal proportions of a feces inoculum mixture obtained from at least three healthy donors. The feces collection was approved by the Ethics Committee of the university hospital of the Friedrich Schiller University Jena, Germany (No. 4625-12/15). Written informed consent was obtained from all subjects. After adjusting the pH-values of all batch samples to 6.5, in vitro fermentation was performed for 24 h in an anaerobic atmosphere at 37 °C. After 24 h, the fermentation was stopped by transfer of the samples to ice, the pH value of each sample was measured, and fermentation supernatants (FS) were obtained by centrifugation [30]. Prior to usage in cell culture experiments, FS were subjected to sterile filtration (Filtropur S, 0.22 µm, Sarstedt AG & Co. KG, Nümbrecht, Germany). For treatment of cells, FS were diluted in cell culture medium to final concentrations.

### 2.3. Cell Culture

LT97 cells (a kind gift from Professor B. Marian, Institute for Cancer Research, University of Vienna, Austria) are cells of an early colon adenoma in the premalignant stage of tumor development. Therefore, this cell line was used as a model of an early stage of colon carcinogenesis. The cell line was prepared from colon microadenoma of a patient suffering from hereditary familiar polyposis coli [28]. The cells were grown in MCDB-302 cell culture medium (PAN Biotech, Berlin, Germany) containing 20% L-15 Leibovitz medium (PAN Biotech, Berlin, Germany), 2 mmol/L L-glutamine (PAN Biotech, Berlin, Germany) and the following supplements: 2% fetal calf serum (PAN Biotech, Berlin, Germany), 10 μg/mL insulin (PAN Biotech, Berlin, Germany), 0.2 nmol/L triiodo-l-thyronine (Merck, Darmstadt, Germany), 2 μg/mL transferrin (PAN Biotech, Berlin, Germany), 1 μg/mL hydrocortisone (Merck, Darmstadt, Germany), 5 nmol/L sodium selenite (Merck, Darmstadt, Germany), 30 ng/mL epidermal growth factor (PAN Biotech, Berlin, Germany), and 50 μg/mL gentamycin (PAN Biotech, Berlin, Germany). Cells of passages 7–28 were used for cell culture experiments.

The human colon adenocarcinoma cell line HT29 (American Type Culture Collection no. HTB-38) was used as a model of an advanced stage of colon carcinogenesis. The origin, properties and cell culture conditions of this cell line have been described previously [34]. In brief, the cells were grown in Dulbecco’s modified Eagle’s medium (PAN Biotech, Berlin, Germany) supplemented with 10% fetal calf serum (PAN Biotech, Berlin, Germany) and 1% penicillin/streptomycin (PAN Biotech, Berlin, Germany). Cells from passages 14–38 were used for experiments.

Both cell lines were maintained at 37 °C in a humidified incubator (5% CO_2_, 95% humidity). Mycoplasma tests (MycoAlert Mycoplasma Detection Kit, Lonza, Switzerland) were performed in regular intervals to exclude contamination with mycoplasma.

### 2.4. Growth Inhibition—DAPI Assay

Growth inhibitory effects of FS of the different fibers and butyrate on LT97 and HT29 cells were analyzed using the DAPI (4′,6-diamidino-2-phenylindol) assay as described previously [35]. Cells were grown to a confluence of approximately 90%. LT97 cells were harvested from 75 cm^2^ cell culture flasks with 5 mL PBS/EDTA (ethylenediaminetetraacetate, 137 mM NaCl, 2.7 mM KCl, 1.5 mM KH_2_PO_4_, 8.1 mM Na_2_HPO_4_, 2.0 mM Na_2_EDTA·2H_2_O, in aqua bidest., pH 7.3). The cell suspension was diluted 1:20 in cell culture medium and 100 µL per well were seeded into 96-well plates. LT97 cells should be seeded as aggregates to ensure cell adhesion. Therefore, the preparation of a single-cell suspension and determination of the cell number was not possible. For experiments with HT29 cells, cells were harvested with 5 mL trypsin/EDTA (PAN Biotech, Berlin, Germany) and 7.5 × 10^4^ cells per well were seeded into 96-well plates. Cells from both cell lines were grown for 48 h (to a confluence of approx. 50–60%) before treatment with 2.5%, 5%, 10%, and 20% of FS (diluted in cell culture medium) as well as 2 mM, 4 mM, and 8 mM butyrate (LT97 cells) or 2.5 mM, 5 mM and 10 mM butyrate (HT29 cells) for 24 h with or without 5 mM NAC (*N*-acetyl-cysteine, Sigma-Aldrich Inc., St. Louis, MO, USA), respectively. For HT29 cells, higher concentrations of butyrate were chosen because of a lower sensitivity regarding butyrate mediated growth inhibitory effects compared to LT97 cells [36]. After treatment, the incubation medium was discarded and cells were fixed with 100 µL methanol (Carl Roth GmbH & Co. KG, Karlsruhe, Germany) for 5 min. Next, methanol was removed and evaporated, and cells were stained with 100 µL of DAPI solution (20 µM in PBS, 30 min, 37 °C, Sigma-Aldrich Inc., St. Louis, MO, USA). The relative cell numbers were calculated from the blank-corrected results based on the medium control, which was set to 100%, after recording the fluorescence at Ex/Em of λ = 360/465 nm (SpectraFluor Plus, Tecan Germany, Crailsheim, Germany). The growth inhibition rates were calculated by subtracting the relative cell number of FS/butyrate (^+^NAC) treated cells from medium (^+^NAC) treated cells in accordance with Wang et al. [25].

### 2.5. ROS Formation—DCF Assay

The detection of ROS as a marker for oxidative stress was measured using the DCF assay as described by Pelka et al. [37] with slight modifications. In brief, LT97 and HT29 cells were seeded into black 96-well plates with clear bottom as described in Section 2.4. The cell culture medium was removed, and cells were washed twice with PBS^++^ (containing Ca^2+^ and Mg^2+^). Subsequently, cells were incubated with 100 µL of a 20 µM DCFH-DA solution (2′,7′-dichlorofluorescin-diacetate, in PBS^++^, Sigma-Aldrich Inc., St. Louis, MO, USA) for 30 min at 37 °C. DCFH-DA was removed and cells were washed again twice with PBS^++^ before treatment with 2.5%, 5%, 10%, and 20% of FS as well as different concentrations of butyrate with or without 5 mM NAC as well as with the positive controls (2 mM H_2_O_2_, 10 mM Tert-butyl hydroperoxide, TBH) for up to 24 h at 37 °C. The fluorescence intensity was measured in 30 min intervals up to 3 h and after 6 h and 24 h at Ex/Em of λ = 485/535 nm (Synergy 2, Biotek Instruments GmbH, Bad Friedrichshall, Germany).

### 2.6. Protein Expression—Western Blotting

Protein expression of p21, Cyclin D2, PARP (full length and 89 kDA fragment), Bid (full length), and GPx2 was measured after SDS-PAGE using Western blot analyses. For this purpose, LT97 cells were harvested from 75 cm^2^ cell culture flasks with 5 mL PBS/EDTA, the whole cell suspension was diluted in 7 mL cell culture medium and 200 µL per well were seeded into 6-well plates. Due to aggregate formation, it was not possible to count LT97 cells. HT29 cells were harvested with 5 mL trypsin/EDTA and 4 × 10^5^ cells per well were seeded into 6-well plates. Both cell lines were grown to a confluence of 50–60% and treated with 5% FS obtained from the different fiber sources as well as different concentrations of butyrate (LT97 cells: 0.5–8 mM, HT29 cells: 2.5–10 mM) both with or without 5 mM NAC for 24 h. Preparation of cell lysates, SDS-PAGE, and Western blotting was performed as described previously [38]. The following primary antibodies were used for Western blot experiments: mouse anti-p21 (1:500, Cell Signaling Technology, Beverly, MA, USA), rabbit anti-cyclin D2 (1:1000, Cell Signaling Technology, Beverly, MA, USA), rabbit anti-PARP (1:500, Cell Signaling Technology, Beverly, MA, USA), rabbit anti-Bid (1:500, Cell Signaling Technology, Beverly, MA, USA), rabbit anti-GPx2 (1:1000, Abcam, Cambridge, UK), rabbit anti-GAPDH (1:5000, Abcam, Cambridge, UK), mouse-anti-GAPDH (1:5000, Abcam, Cambridge, UK). Furthermore, the following secondary antibodies were used: IRDye 800 CW donkey anti-mouse IgG, IRDye 680 LT goat anti-rabbit IgG, IRDye 680 CW donkey anti-mouse IgG, and IRDye 800 LT goat anti-rabbit IgG (1:15,000) (LI-COR Biosciences GmbH, Bad Homburg, Germany). For visualization of protein bands, the Odyssey system (LI-COR Biosciences GmbH, Bad Homburg, Germany) was used. Results are expressed as fold changes in relation to the medium control and the NAC-treated medium control which were set to 1, respectively. Representative images of protein bands are shown in Supplemental Figures S1 and S2.

### 2.7. Statistical Analyses

Unless stated otherwise, means and standard deviations of at least three independent experiments were calculated and statistical differences were analyzed as follows. Relative cell numbers: significant differences between cells treated with butyrate or FS and medium were obtained by two-way ANOVA. Growth inhibition rates: significant differences between cells treated with different concentrations of butyrate or FS (^−/+^NAC) were obtained by one-way ANOVA. Relative ROS induction: significant differences between cells treated with butyrate or FS and medium and significant differences between cells treated with different concentrations of butyrate and FS (^−/+^NAC) were obtained by two-way ANOVA. Protein expression: significant differences between cells treated with different concentrations of butyrate or with different FS (^−/+^NAC) and significant differences compared to medium treated cells were obtained by two-way ANOVA. In all cases, the F-test according to Ryan–Einot–Gabriel–Welsh was used as a post-hoc test.

Significant differences between cells treated with butyrate/FS alone and cells treated with butyrate/FS and NAC as well as between cells treated with medium and positive controls were obtained by unpaired Student’s *t*-test.

Comparison to the medium control was performed using raw data and for comparison of other groups normalized data was used. Statistical analyses were performed using the SPSS Statistics software version 26 (IBM Corporation, Armonk, NY, USA).

## 3. Results

### 3.1. Impact of FS and Butyrate on LT97 Cell Growth

**Figure 1:**
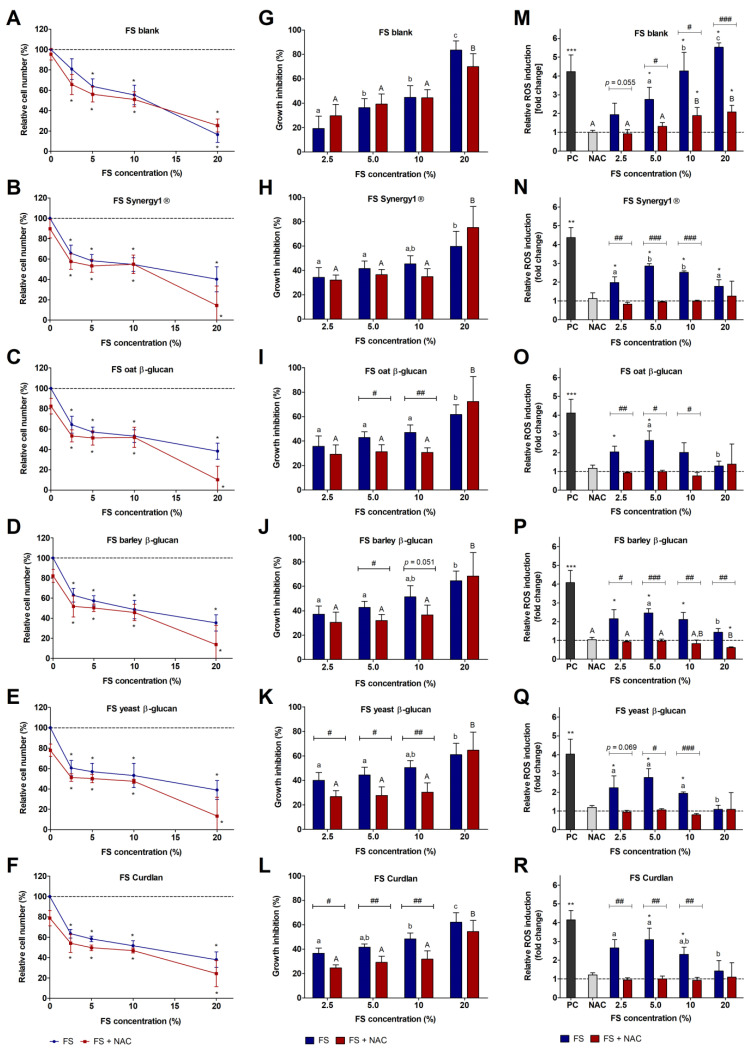
Relative number (%) of LT97 colon adenoma cells (**A**–**F**), growth inhibition rate (%) of LT97 cells (**G**–**L**), and relative ROS induction (fold change) in LT97 cells (**M**–**R**) after treatment with different concentrations (2.5%, 5%, 10%, and 20%) of fermentation supernatants (FS) obtained from the blank control and different dietary fiber samples (Synergy1^®^, oat β-glucan, barley β-glucan, yeast β-glucan, Curdlan) and co-treatment with 5 mM NAC (*N*-acetyl-cysteine) for 24 h. Relative cell numbers were calculated on the basis of the medium control (dashed line) (**A**–**F**), which was set at 100% (mean ± SD, *n* = 4). Growth inhibition rates (%) (**G**–**L**) were calculated by subtraction of the relative cell numbers from the respective medium control (mean + SD, *n* = 4). Relative ROS induction (fold change) (**M**–**R**) was calculated on the basis of the medium control, which was set at 1 (dashed line) (mean + SD, *n* = 3). Significant differences are indicated as follows: compared to medium treated cells (* *p* < 0.05), between cells treated with different concentrations of FS (blank and dietary fibers) without NAC (^−^NAC: ^a–c^ *p* < 0.05, different lowercase letters represent significantly different results) and with NAC (^+^NAC: ^A,B^ *p* < 0.05, different uppercase letters represent significantly different results), between cells treated with FS alone and cells treated with FS and NAC (^#^ *p* < 0.05, ^##^ *p* < 0.01, ^###^ *p* < 0.001) and between cells treated with the positive control (PC = 2 mM H_2_O_2_) and medium (** *p* < 0.01, *** *p* < 0.001).

Treatment of LT97 colon adenoma cells with increasing concentrations of fiber FS resulted in a significant reduction of relative cell numbers in comparison to cells treated with medium (Figure 1B–F). Comparable relative cell numbers were detected after treatment with the blank FS (Figure 1A). In comparison, co-treatments of blank FS and NAC as well as fiber FS and NAC resulted in slightly lower relative cell numbers (Figure 1A–F). Since NAC affected LT97 cell growth, the growth inhibition rates were calculated in relation to the medium control and medium control ^+^NAC for FS treatment and FS ^+^NAC co-treatment (Figure 1G–L), respectively. Here, in general, higher growth inhibitory effects were detected after treatment of cells with fiber FS alone than with fiber FS and NAC together.

Incubation with lower concentrations of the FS from oat- and barley-β-glucan (5%, 10%) as well as yeast-β-glucan and Curdlan (2.5%, 5%, 10%) resulted in significantly lower inhibition of cell growth when applied in combination with NAC than the FS treatment alone. In contrast, no differences between FS treatment and FS and NAC co-incubation were detected for the blank FS and the FS of Synergy1^®^ (Figure 1G,H) and higher concentrations of the other FS (20%).

**Figure 2:**
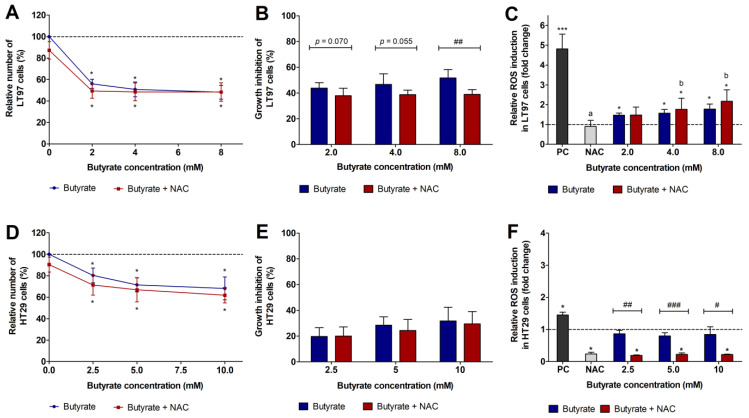
Relative cell number (%) (**A**), growth inhibition rate (%) (**B**) and relative ROS induction (fold change) (**C**) of LT97 cells after treatment with different concentrations (2 mM, 4 mM, 8 mM) of butyrate and co-treatment with 5 mM NAC (*N*-acetyl-cysteine) and relative cell number (%) (**D**), growth inhibition rate (%) (**E**), and relative ROS induction (fold change) (**F**) of HT29 cells after treatment with different concentrations (2.5 mM, 5 mM, 10 mM) of butyrate and co-treatment with NAC for 24 h. Relative cell numbers were calculated on the basis of the medium control (dashed line) (**A**,**D**), which was set at 100% (mean ± SD, *n* = 4). Growth inhibition rates (%) (**B**,**E**) were calculated by subtraction of the relative cell numbers from the respective medium control (mean + SD, *n* = 4). Relative ROS induction (fold change) (**C**,**F**) was calculated on the basis of the medium control, which was set at 1 (dashed line) (mean + SD, *n* = 3). Significant differences are indicated as follows: compared to medium treated cells (* *p* < 0.05), between cells treated with different concentrations of butyrate (^−/+^NAC: ^a,b^ *p* < 0.05, different letters represent significantly different results), between cells treated with butyrate alone and cells treated with butyrate and NAC (^#^ *p* < 0.05, ^##^ *p* < 0.01, ^###^ *p* < 0.001) and between cells treated with the positive control (PC = 2 mM H_2_O_2_) and medium (* *p* < 0.05, *** *p* < 0.001).

Similarly, treatment of cells with different concentrations of butyrate as well as butyrate and NAC resulted in significantly lower relative cell numbers than treatment with the medium control (Figure 2A). The growth inhibition rates calculated in comparison to the respective controls were partly significantly higher after incubation with butyrate than after co-treatment with butyrate and NAC (Figure 2B).

### 3.2. Impact of FS and Butyrate on ROS Induction in LT97 Cells

In general, treatment of LT97 cells with FS obtained from the different dietary fiber samples for 24 h resulted in significantly increased ROS levels (Figure 1M–R) compared to medium-treated cells. Treatment of cells with increasing concentrations of the blank FS also led to increasing ROS levels. Significantly higher levels of ROS compared to the medium control and a dose-dependent effect were detected for treatments with 5%, 10%, and 20% blank FS. In comparison, simultaneous treatment of LT97 cells with 5 mM NAC resulted in significantly lower levels of ROS. The FS obtained from Synergy1^®^ and the different glucans also induced the production of ROS in LT97 cells, but to a lesser extent than the blank FS. Here, in general, significant higher levels of ROS compared to the medium control were measured after treatment with lower concentrations of 2.5–10% FS. These levels were also significantly higher than that detected after treatment with the highest FS concentration. The simultaneous treatment of cells with NAC reduced the fiber FS-induced ROS formation down to the level of the medium control and thus resulted in a significant reduction of ROS compared to the treatments with the respective concentrations of the sole fiber FS. Furthermore, a time-dependent effect on ROS-formation was observed (Supplemental Figure S3).

Treatment of LT97 cells with 2, 4, and 8 mM butyrate increased ROS levels 1.5 ± 0.1-fold, 1.6 ± 0.2-fold, and 1.8 ± 0.3-fold, respectively (Figure 2C). A significant induction compared to the medium control was detected for treatment of cells with 8 mM butyrate. The simultaneous treatment of cells with butyrate and 5 mM NAC led to a similar ROS formation as the treatment with butyrate alone. In general, the incubation of cells with NAC alone did not alter ROS formation in comparison to the medium control, while the positive control H_2_O_2_ (2 mM) significantly enhanced intracellular ROS (4.3 ± 0.6-fold, mean value of all tests). Results of ROS induction over time are presented in Supplemental Figure S4.

### 3.3. Impact of FS and Butyrate on HT29 Cell Growth

**Figure 3:**
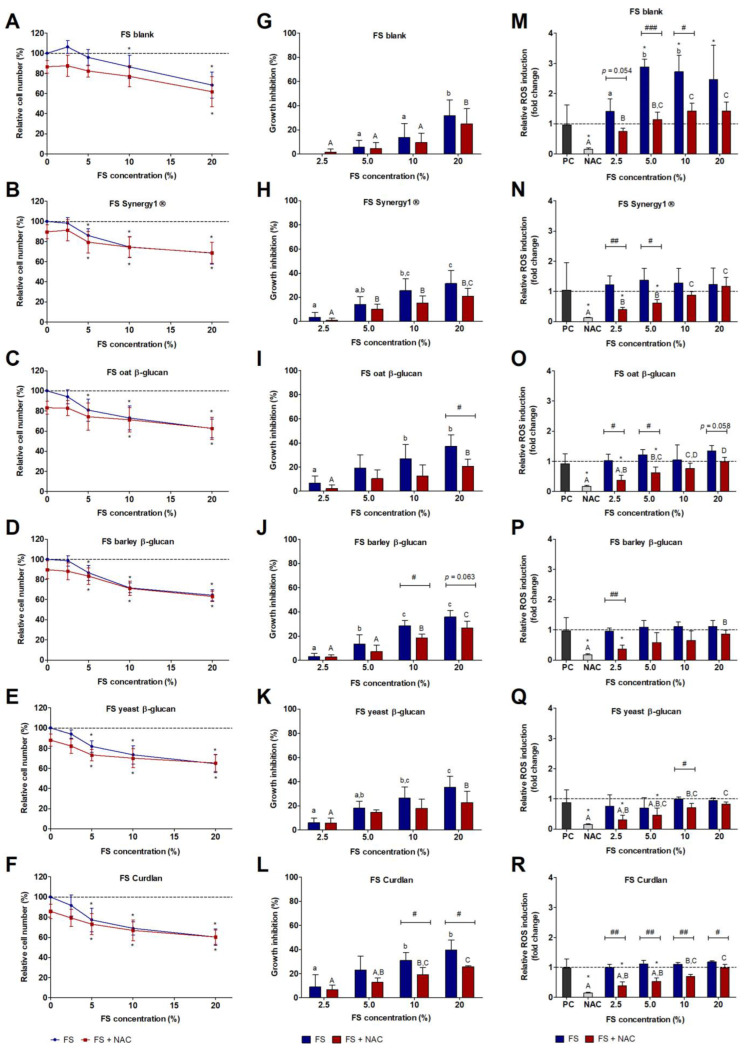
Relative number (%) of HT29 colon carcinoma cells (**A**–**F**), growth inhibition rate (%) of HT29 cells (**G**–**L**), and relative ROS induction (fold change) in HT29 cells (**M**–**R**) after treatment with different concentrations (2.5%, 5%, 10%, and 20%) of fermentation supernatants (FS) obtained from the blank control and different dietary fiber samples (Synergy1^®^, oat β-glucan, barley β-glucan, yeast β-glucan, Curdlan) and co-treatment with 5 mM NAC (*N*-acetyl-cysteine) for 24 h. Relative cell numbers were calculated on the basis of the medium control (dashed line) (**A**–**F**), which was set at 100% (mean ± SD, *n* = 4). Growth inhibition rates (%) (**G**–**L**) were calculated by subtraction of the relative cell numbers from the respective medium control (mean + SD, *n* = 4). Relative ROS induction (fold change) (**M**–**R**) was calculated on the basis of the medium control, which was set at 1 (dashed line) (mean + SD, *n* = 3). Significant differences are indicated as follows: compared to medium treated cells (* *p* < 0.05), between cells treated with different concentrations of FS (blank and dietary fibers) without NAC (^−^NAC: ^a–c^ *p* < 0.05, different lowercase letters represent significantly different results) and with NAC (^+^NAC: ^A–C^ *p* < 0.05, different uppercase letters represent significantly different results), between cells treated with FS alone and cells treated with FS and NAC (^#^ *p* < 0.05, ^##^ *p* < 0.01, ^###^ *p* < 0.001).

Treatment of HT29 cells with different concentrations of the FS of the various fiber sources resulted in a similar and partly dose-depended decrease of relative cell numbers. Compared to the incubation with the medium control, significantly lower numbers of HT29 cells were detected after treatments with 5–20% fiber FS or with 5–20% fiber FS and NAC (Figure 3B–F). In comparison, slightly higher cell numbers were detected after treatment with the blank FS (Figure 3A). In general, combined treatment with NAC and medium, 2.5% or 5% FS, respectively, resulted in lower cell numbers than treatment with medium and the respective FS alone. Again, higher growth inhibitory effects were detected after treatment of cells with fiber FS alone than with combined fiber FS and NAC treatment (Figure 3H–L). In comparison, the blank FS induced slightly lower growth inhibitory effects (Figure 3G). A significant reduction of growth inhibitory effects upon co-treatment with NAC compared to fiber FS treatment alone was detected for the FS of oat β-glucan, barley β-glucan, and Curdlan (Figure 3I,J,L).

Treatment of HT29 cells with butyrate (Figure 2D) resulted in a similar growth reduction as the fiber FS. Here, cell numbers were significantly lower than medium treated cells after incubation with different concentrations of butyrate as well as butyrate and NAC. The calculated growth inhibition compared to the respective controls ranged between 19.7 ± 6.9% and 31.8 ± 10.5% after incubation with 2.5 mM, 5 mM, and 10 mM butyrate, and similarly, between 19.1 ± 5.6% and 28.6 ± 7.9% after co-treatment with butyrate and NAC (Figure 2E).

### 3.4. Impact of FS and Butyrate on ROS Induction in HT29 cells

After 24 h of treatment, the blank FS induced a significant increase in ROS formation compared to the medium control (5% FS: 2.9 ± 0.3-fold, 10% FS: 2.7 ± 0.5-fold, 20% FS: 2.5 ± 1.1-fold). In comparison, treatment with NAC (0.2 ± 0.0-fold) alone and in combination with the blank FS (5% FS: 1.1 ± 0.2-fold, 10% FS: 1.4 ± 0.3-fold) resulted in significantly lower levels of ROS than medium treated cells or cells treated with blank FS alone (Figure 3M). The FS obtained from the different fiber sources had no effect on ROS formation in HT29 cells at this time point. Nevertheless, NAC significantly reduced ROS levels (0.2 ± 0.0-fold, on average) compared to the medium controls and partly also in comparison to the respective FS treatments (Figure 3N–R). Furthermore, for some FS, a slight time-dependent effect was observed for ROS formation. For example, treatment of HT29 cells with increasing concentrations of the blank FS resulted in an increase of ROS levels in an interval of 0–6 h (Supplemental Figure S5). The FS (20%) of oat β-glucan and Curdlan induced a 2.5 ± 0.6-fold and 1.9 ± 0.2-fold increase after 3 h and a 2.1 ± 0.3-fold and 1.8 ± 0.2-fold increase in ROS levels after 6 h, respectively. In general, ROS levels remained unchanged after treatment with FS obtained from Synergy1^®^ and the different β-glucans and treatment with 5 mM NAC resulted in a considerable reduction in ROS formation with even lower values than detected after treatment with the medium control. However, co-incubation with increasing concentrations of FS reverted this effect in a dose-dependent manner (Supplemental Figure S5).

Treatment of HT29 cells with different concentrations of butyrate resulted in similar ROS levels (0.8 ± 0.1-fold, on average) as the medium control (Figure 2F). In comparison, significantly lower ROS levels were calculated after incubation with NAC or with butyrate and NAC (0.2 ± 0.0-fold, on average). Similar results were obtained after measurement of ROS over time (Supplemental Figure S4).

### 3.5. Impact of FS and Butyrate on the Expression of Cell Cycle-, Apoptosis-, and Antioxidant-Relevant Proteins in LT97 Cells

**Figure 4:**
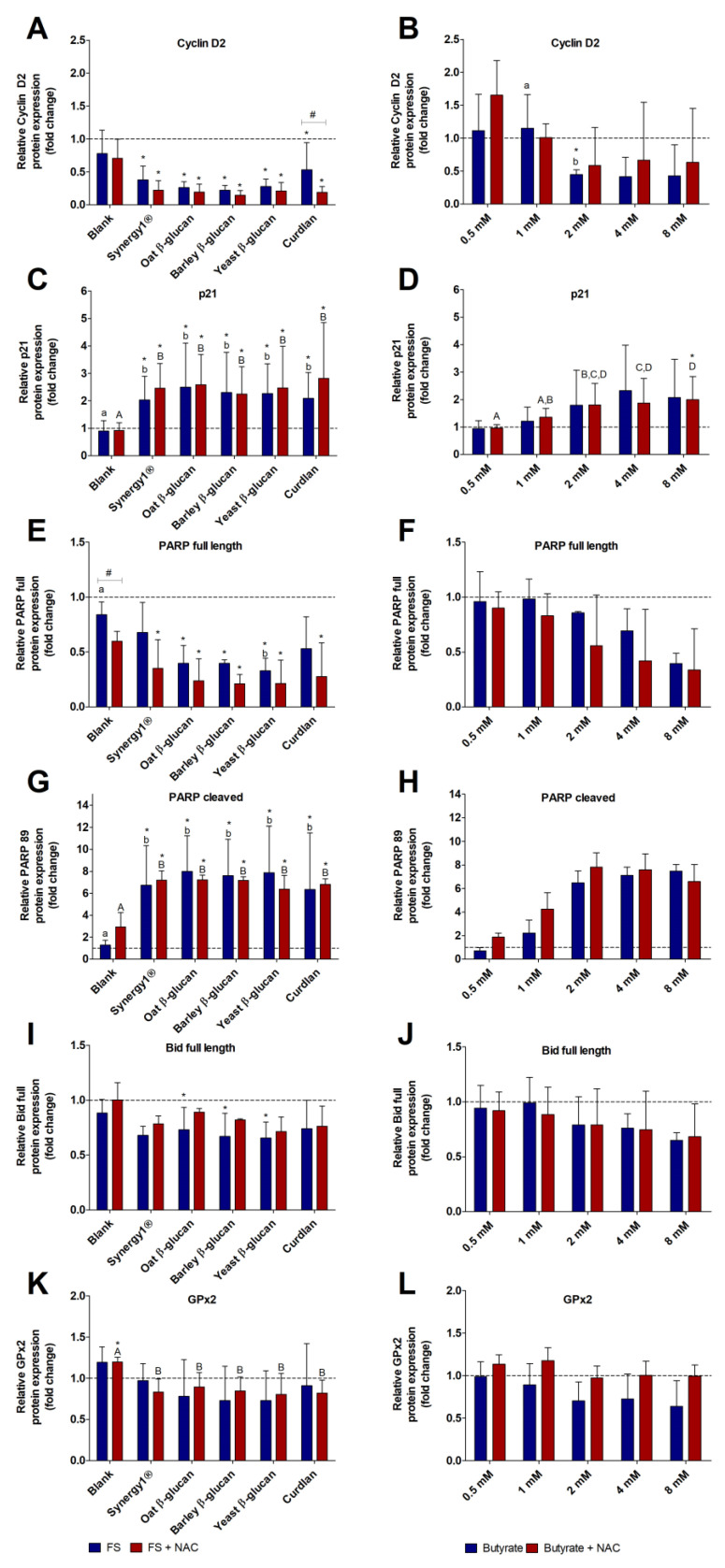
Relative protein expression of Cyclin D2 (**A**,**B**), p21 (**C**,**D**), full-length PARP (**E**,**F**), cleaved PARP (89 kDa) (**G**,**H**), full-length Bid (**I**,**J**), and GPx2 (**K**,**L**) in LT97 cells after treatment with FS (5%) obtained from the blank control and different dietary fiber samples (Synergy1^®^, oat β-glucan, barley β-glucan, yeast β-glucan, Curdlan) and co-treatment with 5 mM NAC (*N*-acetyl-cysteine) (left panel) as well as with different concentrations (0.5 mM, 1 mM, 2 mM, 4 mM, 8 mM) of butyrate and co-treatment with 5 mM NAC (right panel) for 24 h. Results are presented as fold changes based on a medium control, which was set 1 (mean + SD, *n* = 3, (**H**) *n* = 2). Significant differences are indicated as follows: compared to medium-treated cells (* *p* < 0.05), between cells treated with different concentrations of butyrate or with different FS without NAC (^−^NAC: ^a,b^ *p* < 0.05, different lowercase letters represent significantly different results) and with NAC (^+^NAC: ^A–D^ *p* < 0.05, different uppercase letters represent significantly different results), and between cells treated with butyrate/FS alone and cells treated with butyrate/FS and NAC (^#^ *p* < 0.05).

FS (5%) obtained from different β-glucans and Synergy1^®^ significantly reduced Cyclin D2 protein levels (0.3 ± 0.2-fold, on average) in comparison to the medium control, while the blank FS had no impact on Cyclin D2 expression. Simultaneous treatment with NAC had no adverse effect on FS-mediated reduction of Cyclin D2 protein expression (Figure 4A). Treatment of LT97 with 2–8 mM butyrate resulted in an average decrease of Cyclin D2 protein expression of 0.4 ± 0.3-fold compared to medium treated cells (Figure 4B). Here, significant differences in comparison to medium treated cells were detected for the treatment with 2 mM butyrate. Simultaneous treatment with NAC partly led to slightly higher Cyclin D2 protein levels than treatment with butyrate alone.

Treatment of cells with the blank FS resulted in p21 expression that was comparable to medium-treated cells (Figure 4C). Compared to the treatment with the blank FS and medium control, all FS obtained from the different dietary fiber samples and co-incubation with NAC led to significant increase of p21 protein levels (2.2 ± 1.0-fold and 2.5 ± 1.2-fold, respectively, means of all treatments). Treatment of LT97 cells with increasing concentrations of butyrate resulted in increased p21 protein expression levels (up to 2.3 ± 1.7-fold, 4 mM butyrate), which did not reach statistical significance due to large standard deviations (Figure 4D). However, co-treatment with 8 mM butyrate and 5 mM NAC significantly enhanced p21 expression compared to the medium control. Nevertheless, NAC had no effect on butyrate mediated increase of p21 protein levels.

PARP expression was significantly reduced by nearly all FS obtained from the different fiber samples, while the blank FS did not alter PARP expression in comparison to the medium control (Figure 4E). Compared to the treatment with the blank FS, a significant reduction of PARP was detected after treatment with the FS obtained from yeast β-glucan. Furthermore, the protein expression of full-length PARP was dose-dependently reduced by butyrate resulting in the lowest expression after treatment with 8 mM butyrate and 8 mM and 5 mM NAC (Figure 4F). Co-incubation with NAC resulted in slightly stronger reductions of PARP expression than treatment with butyrate or fiber FS alone.

The reduction of full length PARP by FS of the fiber samples and butyrate was associated with a clear increase in cleaved PARP (Figure 4G,H). Significantly higher protein levels of cleaved PARP were detected in LT97 cells treated with FS of the fiber samples (7.3 ± 3.4-fold, on average) than in cells treated with the blank FS (1.3 ± 0.4-fold) or medium control. Treatment with butyrate (8 mM) led to an increase of cleaved PARP levels of up to 7.5 ± 0.6-fold. Co-incubation with NAC had no effect on the butyrate and FS mediated increase of cleaved PARP.

The expression of full-length Bid was slightly decreased after treatment of cells with the different fiber FS with a significant Bid downregulation detected for the FS of oat β-glucan and yeast β-glucan (Figure 4I), while the blank FS had no impact on Bid expression. Similar results were obtained after treatment with increasing concentrations of butyrate (Figure 4J). Furthermore, co-incubation of cells with butyrate or fiber FS and NAC had no significant effect on Bid expression levels in comparison to treatment with fiber FS or butyrate alone.

Treatment of cells with FS of the different fiber samples slightly decreased GPx2 protein expression, while the blank FS did not reduce GPx2 protein expression (Figure 4K). Furthermore, co-treatment with NAC and fiber FS resulted in significantly lower GPx2 levels (0.8 ± 0.2-fold, on average) than treatment with the respective blank FS (1.2 ± 0.1-fold). GPx2 protein levels were insignificantly reduced with increasing concentrations of butyrate, while co-treatment with NAC reversed this effect (Figure 4L). In comparison, representative images of Western blot analyses of the different protein bands are shown in Supplemental Figure S1.

### 3.6. Impact of FS and Butyrate on the Expression of Cell Cycle-, Apoptosis-, and Antioxidant-Relevant Proteins in HT29 Cells

**Figure 5:**
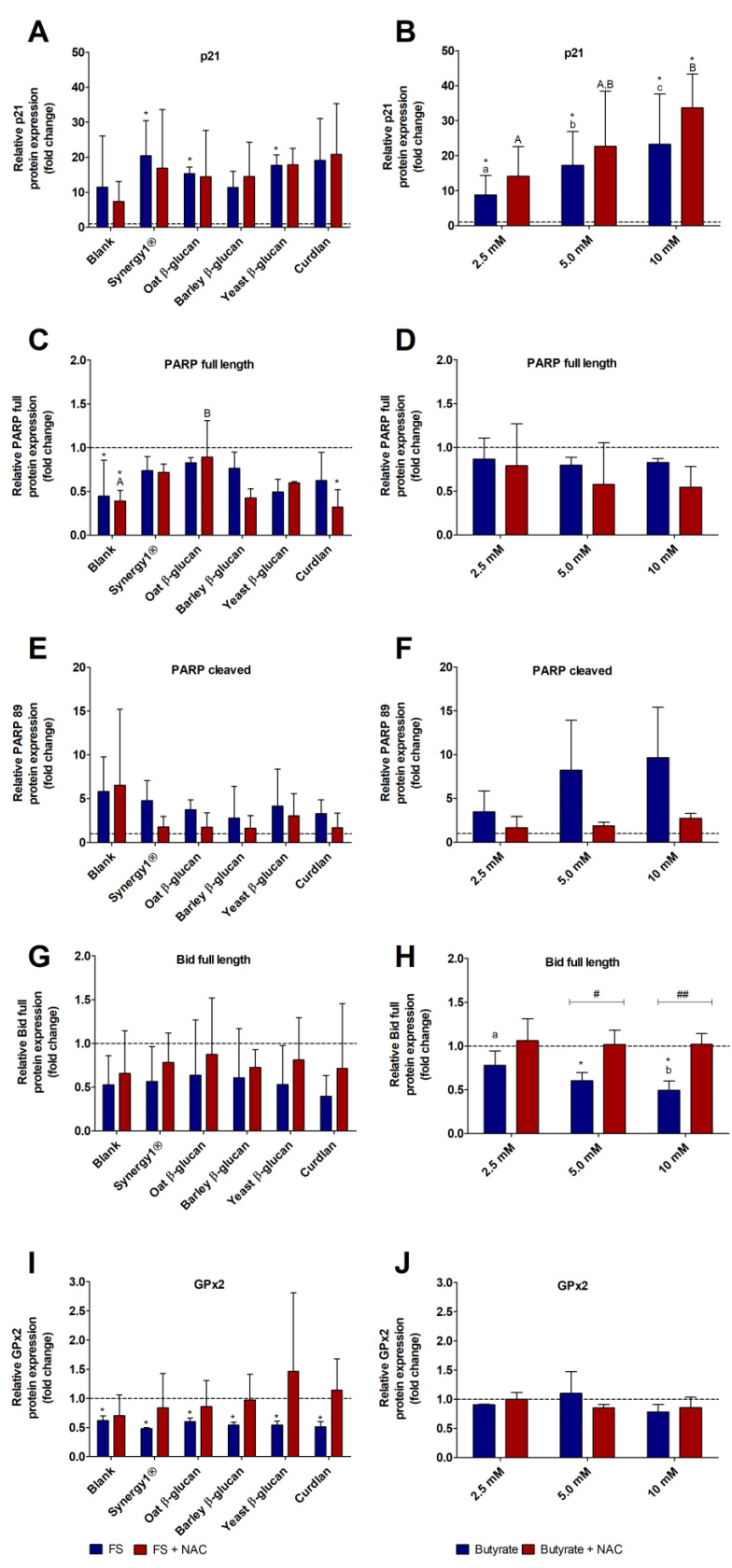
Relative protein expression of p21 (**A**,**B**), full-length PARP (**C**,**D**), cleaved PARP (89 kDa) (**E**,**F**), full-length Bid (**G**,**H**), and GPx2 (**I**,**J**) in HT29 cells after treatment with FS (5%) obtained from the blank control and different dietary fiber samples (Synergy1^®^, oat β-glucan, barley β-glucan, yeast β-glucan, Curdlan) and co-treatment with 5 mM NAC (*N*-acetyl-cysteine) (left panel) as well as with different concentrations (2.5 mM, 5 mM, 10 mM) of butyrate and co-treatment with 5 mM NAC (right panel) for 24 h. Results are presented as fold changes based on a medium control, which was set at 1 (mean + SD, *n* = 3, (**D**,**E**) *n* = 2). Significant differences are indicated as follows: compared to medium-treated cells (* *p* < 0.05), between cells treated with different concentrations of butyrate or with different FS (blank and dietary fibers) without NAC (^−^NAC: ^a–c^ *p* < 0.05, different lowercase letters represent significantly different results) and with NAC (^+^NAC: ^A,B^ *p* < 0.05, different uppercase letters represent significantly different results) and between cells treated with butyrate/FS alone and cells treated with butyrate/FS and NAC (^#^ *p* < 0.05, ^##^ *p* < 0.01).

Treatment of HT29 cells with 5% FS of different fibers led to an increase of p21 levels, which was significant for FS of Synergy1^®^ (20.4 ± 10.1-fold), oat β-glucan (15.3 ± 1.9-fold), and yeast β-glucan (17.7 ± 3.0-fold). A similar, but insignificant, increase of p21 protein levels was observed after treatment with the other fiber FS (Figure 5A). In contrast, the incubation with the blank FS resulted in lowest p21 levels (11.4 ± 14.7-fold). Co-treatment with NAC had no effect on FS-mediated induction of p21 protein expression.

Treatment of HT29 cells with increasing concentrations of butyrate resulted in a significant and dose-dependent increase of p21 protein expression up to 23.3 ± 14.3-fold (10 mM butyrate) (Figure 5B). Co-treatment of butyrate and NAC also led to a dose-dependent increase of p21 protein levels with a significant induction detected for 10 mM butyrate and NAC (33.7 ± 9.7-fold). However, no significant differences in p21 protein expression were detected between butyrate and NAC co-treated cells.

Treatment of cells with FS of the different fiber fractions led to a reduction of full-length PARP expression (0.7 ± 0.2-fold, on average) with the lowest expression detected for yeast glucan (0.5 ± 0.1-fold) (Figure 5C). Significantly lower levels of PARP compared to medium-treated cells were measured after incubation with the blank FS (0.4 ± 0.4-fold), and co-treatments of blank FS and NAC (0.4 ± 0.1-fold) as well as with Curdlan and NAC (0.3 ± 0.2). In comparison, full-length PARP expression in HT29 cells was slightly decreased after treatment with butyrate down to 0.8 ± 0.0-fold (10 mM butyrate) and 0.5 ± 0.2-fold (10 mM butyrate and NAC), but without significant impact of NAC (Figure 5D).

In accordance, levels of cleaved PARP increased after incubation with FS obtained from the different fiber samples (Figure 5E). Here, treatment with the blank FS resulted in a 5.8 ± 3.9-fold increase of cleaved PARP and NAC co-incubation led to a 6.5 ± 8.7-fold increase. In contrast, treatment with the fiber FS resulted in an average increase of 3.7 ± 2.2-fold, while NAC slightly decreased this FS mediated PARP cleavage (2.0 ± 1.4-fold). Furthermore, levels of cleaved PARP increased with increasing concentrations of butyrate (Figure 5F). In contrast, co-treatment with NAC clearly reduced cleaved PARP expression.

The FS of all fibers induced an average reduction of full length Bid of 0.5 ± 0.4-fold and co-incubation with NAC resulted in a slightly higher mean level of Bid of 0.8 ± 0.4-fold (Figure 5G). Protein levels of full-length Bid were significantly reduced after treatment with increasing concentrations of butyrate (Figure 5H). In comparison, co-treatment with NAC abrogated the effects of butyrate.

GPx2 expression was significantly reduced by FS obtained from the blank control and the different fiber samples (0.5 ± 0.1-fold, mean of all treatments), while co-incubation with NAC resulted in elevated levels of GPx2 that were comparable to medium treated cells (1.0 ± 0.7-fold, mean of all treatments) (Figure 5I). In contrast, after treatment of HT29 cells with butyrate or butyrate and NAC together, no changes in GPx2 protein levels were detected in comparison to medium treated cells (Figure 5J). Representative images of Western blot analyses of the different protein bands are shown in Supplemental Figure S2.

## 4. Discussion

The chemopreventive properties of butyrate as one of the main metabolites resulting from dietary fiber fermentation in the colon have been studied intensively. The main mechanism by which butyrate mediates these effects may be its well-documented and proven function as HDI [6,8,9]. Nevertheless, other mechanisms of action might contribute to the HDI function of butyrate. Several studies indicate that the generation of ROS or a change in the redox state might be involved in the induction of apoptosis in colon cancer cells contributing to secondary chemopreventive effects of butyrate [25,26,27,39,40].

ROS play a crucial role in the initiation and promotion of cancer since ROS induce DNA damage and activate pro-oncogenic signaling pathways, promote genomic instability, proliferation and growth of cancer cells, metastasis, and resistance against chemotherapy [41,42]. In general, cancer cells have accelerated ROS levels as well as increased ROS scavenging capacities due to an upregulated antioxidant defense system that maintain ROS levels below a cytotoxic threshold. However, the constantly high levels of ROS promote the susceptibility of cancer cells towards cytotoxic ROS accumulation and excessive ROS formation can result in the induction of cell death for example via apoptotic mechanisms [41,42,43]. Cytotoxic effects of butyrate that are associated with ROS formation have been shown in different types of cancer cells such as bladder cancer cells [24], breast cancer cells [23], hepatic cancer cells [22], or SAS tongue cancer cells [44]. However, at least for colon cancer cells, results from studies investigating ROS-mediated effects by butyrate remain inconclusive. For example, Wang et al. [25] demonstrated that butyrate-mediated inhibition of colon cancer cell growth (HT29, SW480) was associated with a downregulation of Thioredoxin-1 and an increase in ROS levels. In contrast, Domokos et al. [26] concluded from their studies with different HT29 cell lines that elevated ROS levels are the consequence of butyrate-induced cell death and not the trigger. Furthermore, Matthews et al. [27] showed that butyrate altered the redox-state of Caco-2 cells but concluded that elevated ROS levels are not directly associated with apoptotic effects. Therefore, the aim of the present study was to elucidate the potential role of ROS in chemopreventive properties of butyrate as a single metabolite as well as FS of different dietary fibers representing complex mixtures of fermentation products to better simulate and approximate the in vivo situation. Furthermore, different cell types representing different stages of transformation were used in the present study. LT97 cells are characterized by a loss of both alleles of the tumor suppressor gene *APC* and a mutated Ki-*ras* allele, while *p53* remains intact [28,29], thus representing a rather early stage of tumorigenesis. HT29 cells have already a mutation in the tumor suppressor gene *p53* [45], which occurs later in colon tumorigenesis [46]. Therefore, HT29 cells represent a model for a late stage of colon carcinogenesis. Due to these differences, LT97 and HT29 cells might react differently on cellular or metabolic stressors since the sensitivity towards ROS induced cell death seems to be dependent on the p53 status [47]. Overall, this is a unique approach since to our knowledge, the impact of complex mixtures of metabolites resulting from digestion and fermentation of different fibers on ROS formation and their impact on chemoprevention in different types of cancer cells has not yet been investigated.

Most obviously, the results from the present study verified a higher sensitivity of LT97 cells towards butyrate- and FS-mediated growth inhibition than HT29 cells, resulting in lower IC_50_- and IC_25_-values for LT97 cells (Supplemental Table S1). These results are in line with results obtained in former studies investigating the growth inhibitory potential of butyrate on LT97 and HT29 cells. Kautenburger et al. [36] demonstrated a 2-fold higher sensitivity of LT97 cells towards butyrate compared to HT29 cells, which was also the reason to choose lower butyrate concentrations for experiments with LT97 cells in the present study. Furthermore, the authors found a 2-fold higher concentration of butyrate in LT97 cells than in HT29 cells probably due to a higher uptake of butyrate which might result from higher expression levels of the butyrate-transporting monocarboxylate transporter MCT-1. The mRNA and protein expression levels of MCT-1 were found to be decreased during progression from normal to malignant states in colon cells [48]. In the present study, co-treatment of LT97 cells with NAC significantly diminished the butyrate mediated growth inhibition at a concentration of 8 mM butyrate after 24 h. However, NAC did not decrease the significantly enhanced ROS levels observed after treatment with 8 mM butyrate. These results suggest that the butyrate-mediated growth inhibition of LT97 cells is not directly associated with the formation of ROS. In contrast, ROS levels were not affected by 2.5 mM, 5 mM, or 10 mM butyrate in HT29 cells after treatment for 24 h. Though NAC significantly decreased basal ROS levels independently from butyrate treatment, butyrate-mediated growth inhibition of HT29 cells was not affected by NAC. These results are in contrast with results obtained by Wang et al. [25] who demonstrated an increase of ROS in HT29 cells treated with 2.5 mM butyrate for 48 h. Furthermore, growth inhibitory effects of butyrate were significantly diminished by NAC (concentration not stated). Domokos et al. [26] also showed an increase of ROS in HT29R cells after treatment with up to 20 mM butyrate for 48 h. Furthermore, Giardina and Inan [49] found that treatment of HT29 cells with butyrate (concentration not stated) for 20 min increased ROS levels. ROS were significantly decreased after treatment with 25 mM NAC to less than the basal levels. This reduction is comparable to the decrease in basal ROS levels observed in the present study using 5 mM NAC. It cannot be excluded that a higher NAC concentration might have more effects on butyrate mediated growth inhibition in HT29 cells or on ROS production in LT97 cells. Higher concentrations of NAC also reduced ROS levels in LT97 cells immediately after application (Supplemental Figure S6), but the use of higher NAC concentrations was not applicable in this study since NAC exerts cytotoxic effects at higher concentrations for longer incubation times (e.g., 24 h) both in HT29 cells as shown by Amini et al. [50] and in LT97 as shown in Supplemental Figure S7. However, differences regarding the induction of ROS by butyrate and the effects of NAC on butyrate-mediated growth inhibition between the present study and others may be due to different concentrations and time points chosen for experiments. However, the results are difficult to compare due to the partially missing information regarding used concentrations (e.g., butyrate or NAC). In conclusion, the results from the present study indicate no concrete association of butyrate induced ROS formation and growth inhibitory effects in both cell lines for the chosen concentrations and times of treatment.

Furthermore, ROS induction and effects of NAC on growth inhibition were investigated in LT97 and HT29 cells after treatment with FS of different dietary fiber sources. Chemopreventive properties such as growth inhibitory effects of fermentation samples of different fibers or fiber-rich foods such as aleurone [51], bread [35], nuts [52], barley [30], and oat flakes [53] as well as single β-glucans [20] were investigated before and may be mainly attributable to butyrate as one of the main fermentation metabolites via its function as HDI. To our knowledge, the involvement of ROS in FS-mediated chemopreventive effects has not been investigated sufficiently so far. In the present study, different fiber sources were used to address differences in physicochemical properties, which affect solubility and fermentability [54]. Usually, so-called soluble dietary fiber such as Synergy1^®^ as well as oat and barley β-glucan have a higher fermentability than insoluble dietary fiber. Yeast β-glucan and Curdlan are less soluble due to their molecular structure [55], which results in slight differences in their fermentability compared to the other soluble fibers [20]. The highest molar ratio of butyrate was detected after fermentation of Synergy1^®^, followed by β-glucans obtained from barley and oat, and Curdlan and yeast. Furthermore, high levels of propionate were found in the FS of yeast β-glucan and Curdlan. This SCFA also induces chemopreventive effects, but to a lesser extent than butyrate [36,56]. Altogether, the results from a recent study [20] and the present study indicate that differences in the fermentation profile have no measurable impact on growth inhibitory effects of the different fermented fibers.

Again, LT97 cells exhibited a higher sensitivity towards FS mediated growth inhibitory effects than HT29 cells. Growth inhibition in LT97 cells was up to 1.6–4.1-fold and 2.4–6.2-fold higher after treatment with 20% FS and 20% FS and NAC, respectively, than in HT29 cells. At this concentration, the different FS contained between 4.1 mM and 4.7 mM butyrate, but they exhibited considerable stronger growth inhibitory effects, at least in LT97 cells, than butyrate alone in comparable concentrations. Furthermore, also the blank FS, representing the negative control, exerts strong inhibitory effects on LT97 as well as on HT29 cell growth. These effects are comparable to those induced by the FS of the different dietary fibers though it contained the lowest concentration of butyrate (1.3 mM). These results are in line with former studies [20,36,51,56] and indicate that also other compounds of the FS such as propionate [35,36,56] or components of the feces matrix may contribute to growth inhibitory effects. For example, secondary bile acids such as deoxycholic acid induce growth inhibitory effects in colon cancer cells [57,58]. The concentration of secondary bile acids was considerably higher in the blank FS than in the FS obtained from the different fibers [20] and might therefore contribute to the observed effects. As reviewed by Zeng et al. [58], secondary bile acids induce apoptosis mainly via the activation of intrinsic apoptotic pathways including mitochondrial oxidative stress and ROS formation. An increase in ROS levels and apoptosis induced by secondary bile acids was also shown in colon cancer cells [57,59,60]. In accordance, the present study detected the highest levels of ROS in LT97 as well as HT29 cells after treatment with the blank FS. The comparison to treatments with the other FS obtained from Synergy1^®^ and the different β-glucans highlights this observation. Therefore, a contribution of secondary bile acids to the induction of ROS formation and growth inhibition, at least for the blank FS, cannot be excluded. Higher levels of ROS were also detected in LT97 cells after treatment with fiber FS with exception of the highest FS concentration, which might be explainable by the strongly reduced cell numbers.

Co-treatment of LT97 cells with 2.5–10% FS and NAC resulted in significantly lower ROS levels compared to FS treatment alone. Furthermore, co-treatment with NAC resulted in significantly lower growth inhibition rates in LT97 cells compared to treatment with FS alone, at least with FS obtained from the different glucans. Though NAC completely reversed ROS formation to basal levels, it could not completely block growth inhibitory effects induced by the FS. In HT29 cells, no significant increase in ROS formation was observed after incubation with the fiber FS except for the treatment with the blank FS. In addition, the positive control (TBH) failed to induce ROS formation, at least for the chosen time point of 24 h incubation as at earlier treatment time points (3 h, 6 h) a slight increase in ROS levels was observed (Supplemental Figure S5) and a small but significant increase was detected in the butyrate treatment series (Figure 2, Supplemental Figure S4). This low or missing induction of ROS formation might be due to the already enhanced basal ROS levels and antioxidant defense system observed in cancer cells [61]. NAC partially reduced ROS levels far below basal levels similarly to the reduction observed after butyrate treatment. This effect was reversed with increasing fiber FS concentrations. Nevertheless, NAC partly significantly diminished FS induced growth inhibition, but to a lesser extent than in LT97 cells. Similarly, NAC did not completely abolish growth inhibitory effects. These results are in line with other studies investigating the effects of potential ROS contribution to induction of cell death or growth inhibition via butyrate in colon cancer cells as discussed before [26,27]. Zhu et al. [61] for example investigated the impact of ROS on growth of different colon cancer cells and observed no evident dose–response relationship between cellular ROS levels and cytotoxicity. Furthermore, Lee et al. [47] showed that the induction of ROS formation is dependent on the p53 status, which might explain the differences in ROS induction and inhibition of cell growth between HT29 and LT97 cells. Due to the wild type p53 status, LT97 cells might be more susceptible to ROS formation as well as to NAC induced reduction of cell growth inhibition than p53 altered HT29 cells. Therefore, the results of the present study are difficult to interpret. Nevertheless, a contribution of ROS to the growth inhibitory effects of the different FS including the blank FS cannot be excluded at least for LT97 cells. However, FS represent complex mixtures of different compounds such as SCFA or bile acids, which might act synergistically or interact via different pathways [58,62,63].

To further elucidate underlying mechanisms of growth inhibition induced by butyrate and the fiber FS as well as a potential contribution of ROS, the expression of proteins involved in cell cycle and apoptosis as well as antioxidant defense was investigated. Here, the expression of Cyclin D2 was only examined in LT97 cells since it is not expressed in HT29 cells [64]. This protein expression was reduced by butyrate and FS of all investigated fibers. In contrast, protein levels of p21 were increased in LT97 cells by butyrate and fiber FS. In comparison, the increase in p21 protein levels was more pronounced in HT29 cells after treatment with butyrate and partly by fiber FS. These results are in line with former studies investigating the effects of butyrate and FS of different dietary fiber rich foods [38,65]. The reduction of Cyclin D2 and the induction of p21 protein expression may be mainly attributable to butyrate. For example, studies indicated that butyrate regulates the expression of cell cycle-relevant genes, such as p21 and Cyclins via its function as HDI resulting in cell cycle arrest [8,9,65,66]. Furthermore, butyrate blocks proliferation by stimulation of p21 [67] and reduction of Cyclin D1 [68] protein expression. In the present study, NAC had no impact on the butyrate- or FS-mediated effects on Cyclin D2 and p21 protein expression in both cell lines. These results may indicate that these cell cycle relevant proteins were not affected by ROS. However, for example, results from other studies show that butyrate-mediated induction of apoptosis is regulated by a complex signaling feedback loop involving p21, ROS, and p53 [69] and that the induction of ROS is caused by elevated p21 levels [70].

Furthermore, the cleavage of PARP as a well-accepted marker for apoptotic cells was detected in both cell lines after treatment with butyrate and fiber FS. The increase of levels of cleaved PARP was more pronounced in LT97 cells, particularly after treatment with the fiber FS. Thus, these results indicate that LT97 cells are more sensitive towards butyrate and FS treatment than HT29 cells, which matches the results obtained for growth inhibitory effects. Co-treatment with NAC had no significant impact on butyrate- or FS-mediated PARP cleavage in LT97 cells. Similar results were obtained after co-treatment of HT29 cells with the different fiber FS and NAC. In contrast, a considerable reduction of levels of cleaved PARP was detected after co-treatment of HT29 cells with NAC compared to butyrate treatment alone. A similar impact of NAC on butyrate-induced PARP cleavage was observed by Wang et al. in human bladder cancer cells [24], indicating that ROS production was associated with butyrate-mediated apoptosis and autophagy. Interestingly, in the present study, butyrate-mediated reduction of full-length Bid was also reversed by NAC co-treatment in HT29 cells, whereas for FS treatment and in LT97 cells, in general, no effect of NAC was observed.

The Bcl-2 family protein Bid is a linker between intrinsic and extrinsic apoptosis, and is synthesized as an inactive zymogen, which is cleaved and thereby activated, e.g., by caspase-8 [71]. Therefore, the observed decrease of full-length Bid in butyrate-treated cells reflects cleavage and activation of pro-apoptotic Bid. A similar decrease of full-length Bid by butyrate was also observed in HCT116 cells [72]. In the present study, butyrate failed to induce ROS formation in HT29 cells, but the NAC-induced decrease of ROS below basal levels might be associated with the observed effects on PARP and Bid. Nevertheless, these effects were not associated with changes in growth inhibition. These results suggest that Bid is activated by a ROS-dependent mechanism, at least in HT29 cells. The Bid activation might therefore be involved in the enhancement of apoptotic triggers as observed in type-2 cells (e.g., hepatocytes, pancreatic β cells, cancer cells) [73], where it is activated by caspase 8. Furthermore, Bid can be cleaved by caspase 2 which can also be activated e.g., by ROS [74]. Interestingly, in a recent study we demonstrated that butyrate as well as FS of Synergy1^®^ and other fiber-rich bread samples induce caspase 2 activity in LT97 cells [38]. A significant induction of caspase 2 after treatment with FS of the different β-glucans was also observed in the present study (Supplemental Figure S8). In general, different (intrinsic and extrinsic) pathways might be involved in butyrate- and FS-mediated induction of apoptosis as shown in different studies with different colon cancer cells [49,75,76,77,78,79,80,81,82]. Further investigations are necessary to elucidate the role of ROS, Bid, and caspase 2 activation in the butyrate- and FS-mediated induction of apoptosis in colon cancer cells also in relation to the p53 status.

GPx2 is part of the antioxidant defense system of cells and exhibits anti-carcinogenic properties via a protection of ROS induced DNA damage and initiation of carcinogenesis. However, studies indicated that GPx2 may have a divergent role in colon carcinogenesis since it is overexpressed in colon cancer cells and might protect the cancer cells from ROS-induced cell death. Therefore, GPx2 seems to have differential roles depending on the transformation stage of cancer cells [83,84]. Therefore, a downregulation of GPx2 may be associated with a lower risk for colon cancer promotion. In the present study, GPx2 protein expression was slightly reduced by butyrate and FS of fiber in LT97 cells. A more pronounced and significant effect on GPx2 protein expression was observed in HT29 cells after treatment with FS obtained from dietary fibers. Similar results for, e.g., GPx1 gene expression were obtained in former studies investigating chemopreventive effects of FS generated from different fiber rich sources such as nuts or barley [30,35]. NAC partly reversed the effects induced by butyrate (LT97 cells) or by fiber FS (HT29 cells), but due to high standard deviations, these effects were not significant. GPx2 is a target gene of Nrf2 which is a redox sensitive signaling protein [83,85]. Therefore, it cannot be excluded that ROS contribute to the observed modulation of GPx2 protein expression induced by butyrate or FS.

## 5. Conclusions

Taken together, the results from the present study show that a reduction of ROS is associated with a decrease of growth inhibitory effects, partly of butyrate as well as FS of different dietary fibers in LT97 colon adenoma and HT29 colon carcinoma cells. LT97 cells were more susceptible towards butyrate- and FS-mediated growth inhibition, ROS formation, and the impact of ROS reduction by NAC treatment than HT29 cells. These results might imply a better effectiveness of chemoprevention at earlier stages of colon cancer development. This might offer possibilities for a nutritional intervention to enhance prevention of colon carcinogenesis but also to support therapeutic strategies.

However, no concrete impact of ROS on the butyrate- and FS-induced modulation of Cyclin D2, p21, PARP, Bid, and GPx2 protein expression was observed in LT97 cells. In contrast, in HT29 cells, the cleavage of PARP and activation of Bid by butyrate and the modulation of GPx2 by FS might be associated with intracellular ROS levels. Therefore, a contribution of ROS to butyrate- or FS-mediated growth inhibitory effects cannot be excluded, but the mechanism and involved pathways should be be investigated in further studies. The results may also indicate that the use of antioxidants, such as NAC, interfere with anticancer activities of butyrate and FS, particularly in advanced stages of cancer. Therefore, special care should be taken regarding the use of antioxidants during cancer therapy.

To our knowledge, this is the first study investigating the role of ROS in FS- and butyrate-mediated chemopreventive effects regarding the inhibition of growth of colon cancer cells from different transformation stages and it provides a basis for further mechanistic studies.

## Data Availability

The data presented in the study are available on request from the corresponding author.

## Supplementary Materials

The following supporting information can be downloaded at: https://www.mdpi.com/article/10.3390/cancers15020440/s1, Figure S1: Representative images of Western blot analyses of p21, Cyclin D2, PARP (full length and 89 kDa), full-length Bid, GPx2 and GAPDH (reference protein) protein expression in LT97 cells after treatment with the medium control (MC), different concentrations of butyrate (0.5–8 mM; right panel) and fermentation supernatants (FS) obtained from different dietary fiber samples (FS1: blank control, FS2: Synergy1^®^, FS3: oat β-glucan, FS4: barley β-glucan, FS5: yeast β-glucan, FS6: Curdlan; left panel) and co-treatment with 5 mM *N*-acetyl-cysteine (NAC); Figure S2: Representative images of Western blot analyses of p21, PARP (full length and 89 kDa), full-length Bid, GPx2, and GAPDH (reference protein) protein expression in HT29 cells after treatment with the medium control (MC), different concentrations of butyrate (2.5–10 mM/40 mM; right panel), and fermentation supernatants (FS) obtained from different dietary fiber samples (FS1: blank control, FS2: Synergy1^®^, FS3: oat β-glucan, FS4: barley β-glucan, FS5: yeast β-glucan, FS6: Curdlan; left panel) and co-treatment with 5 mM *N*-acetyl-cysteine (NAC); Figure S3: Relative ROS induction (fold change) in LT97 cells after treatment with different concentrations (2.5%, 5%, 10%, and 20%) of fermentation supernatants (FS) obtained from the blank control (A) and different dietary fiber samples (Synergy1^®^ (B), oat β-glucan (C), barley β-glucan (D), yeast β-glucan (E), Curdlan (F)) and after co-treatment with 5 mM NAC (*N*-acetyl-cysteine) as well as with the PC (positive control, 2 mM H_2_O_2_) for 0 h, 3 h, 6 h, 24 h; Figure S4: Relative ROS induction (fold change) in LT97 cells after treatment with different concentrations (2 mM, 4 mM, 8 mM) of butyrate and with the PC (positive control, 2 mM H_2_O_2_) (A) and relative ROS induction (fold change) in HT29 cells after treatment with different concentrations (2.5 mM, 5 mM, 10 mM) of butyrate and with the PC (10 mM *tert*-butyl hydroperoxide) (B) and co-treatment with 5 mM NAC (*N*-acetyl-cysteine) for 0 h, 3 h, 6 h, 24 h; Figure S5: Relative ROS induction (fold change) in HT29 cells after treatment with different concentrations (2.5%, 5%, 10%, and 20%) of fermentation supernatants (FS) obtained from the blank control (A) and different dietary fiber samples (Synergy1^®^ (B), oat β-glucan (C), barley β-glucan (D), yeast β-glucan (E), Curdlan (F)) and after co-treatment with 5 mM NAC (*N*-acetyl-cysteine) as well as with the PC (positive control, 10 mM *tert*-butyl hydroperoxide) for 0 h, 3 h, 6 h, 24 h; Figure S6: Relative ROS induction (fold change) in LT97 cells after treatment with different concentrations (3.125–100 mM) of NAC (*N*-acetyl-cysteine) and with the PC (positive control, 2 mM H_2_O_2_) for 0 h, 3 h, 6 h, 24 h; Figure S7: Relative number (%) of LT97 colon adenoma cells after treatment with different concentrations (3.125–100 mM) of NAC (*N*-acetyl-cysteine) for 24 h; Figure S8: Relative activities of caspase 2 in LT97 cells after treatment with 2.5% and 5% fermentation supernatant (FS) obtained from the blank control and different dietary fiber samples (Synergy1^®^, oat β-glucan, barley β-glucan, yeast β-glucan, Curdlan) as well as 4 mM butyrate for 24 h (A) and 48 h (B). Table S1: IC_50_- and IC_25_-values of LT97 and HT29 cells after treatment with butyrate and FS obtained from different β-glucans.
