# Supplementary material for: Potential Role of ROS in Butyrate- and Dietary Fiber-Mediated Growth Inhibition and Modulation of Cell Cycle-, Apoptosis- and Antioxidant-Relevant Proteins in LT97 Colon Adenoma and HT29 Colon Carcinoma Cells
Source: Cancers (Basel). 2023 Jan 10;15(2):440. doi: 10.3390/cancers15020440 (PMC9857069; doi:10.3390/cancers15020440)
Supplement: Supplementary file 1 [file cancers-15-00440-s001.zip › cancers-2114755-supplementary.pdf]

Supplementary Material

Potential Role of ROS in Butyrate- and Dietary Fiber-Mediated Growth Inhibition and Modulation of Cell Cycle-, Apoptosis- and Antioxidant-Relevant Proteins in LT97 Colon Adenoma and HT29 Colon Carcinoma Cells

Wiebke Schlörmann *, Christoph Horlebein, Sabine M. Hübner, Elisa Wittwer and Michael Glei

Department of Applied Nutritional Toxicology, Institute of Nutritional Sciences, Friedrich Schiller University Jena, Dornburger Straße 24, 07743 Jena, Germany; wiebke.schloermann@uni-jena.de (W.S.)*

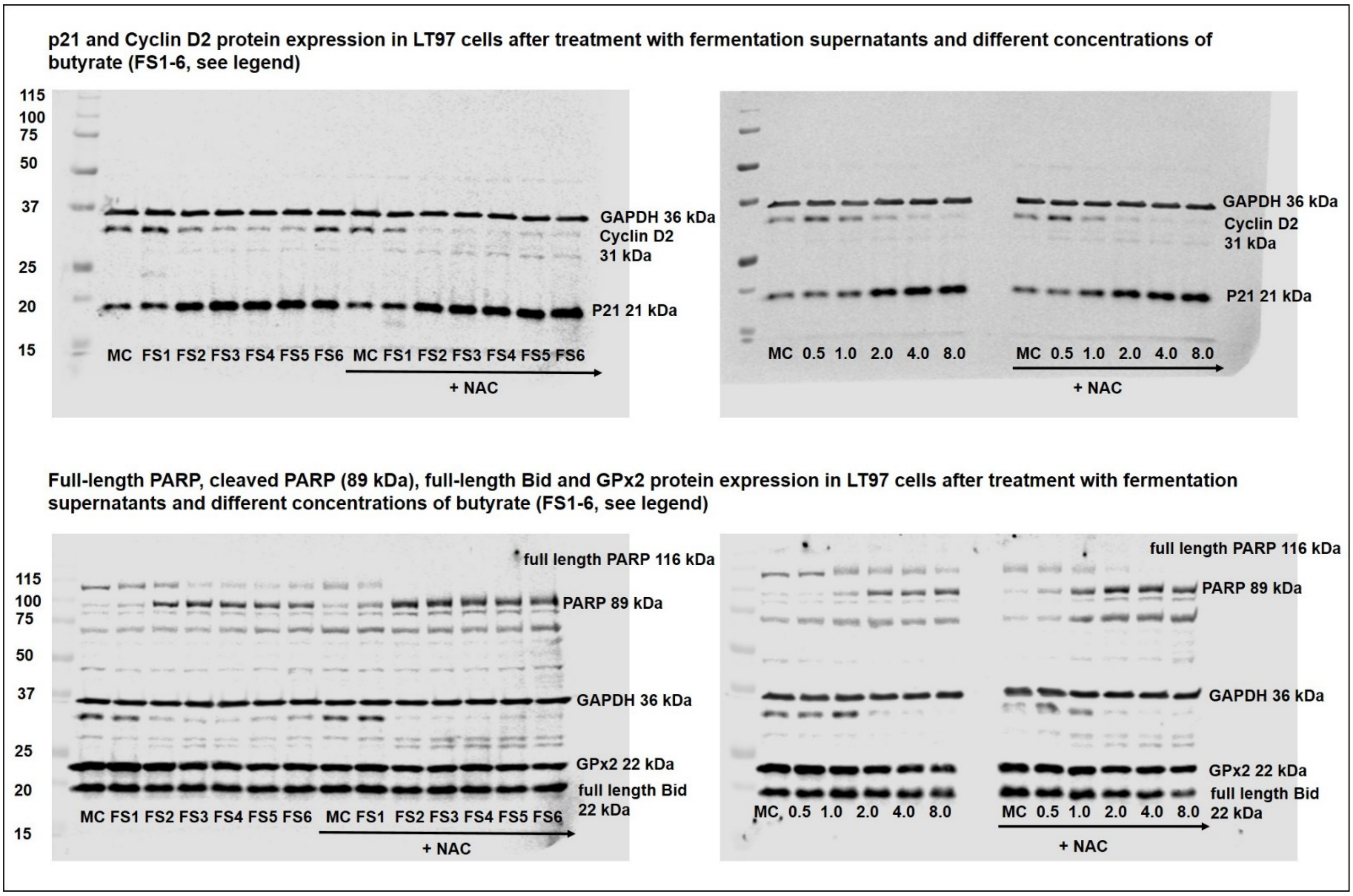

Figure S1. Representative images of Western blot analyses of p21, Cyclin D2, PARP (full length and 89 kDa), full length Bid, GPx2 and GAPDH (reference protein) protein expression in LT97 cells after treatment with the medium control (MC), different concentrations of butyrate (0.5-8 mM; right panel) and fermentation supernatants (FS) obtained from different dietary fiber samples (FS1: blank control, FS2: Synergy1®, FS3: oat β-glucan, FS4: barley β-glucan, FS5: yeast β-glucan, FS6: Curdlan; left panel) and co-treatment with 5 mM *N*-acetyl-cysteine (NAC). Band sizes are predicted molecular weight band sizes indicated by the suppliers of the antibodies. The real band size may vary moderately.

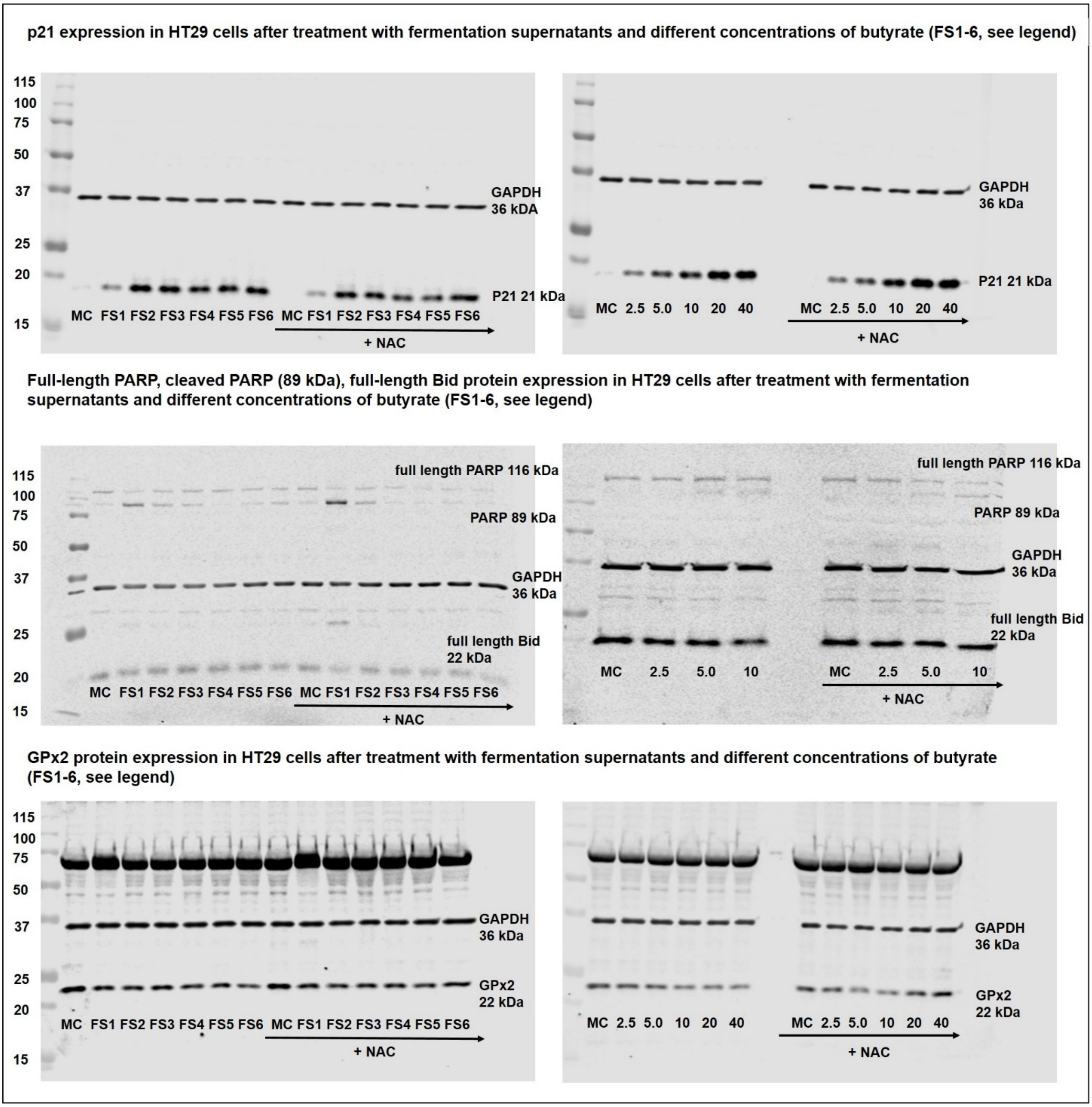

Figure S2. Representative images of Western blot analyses of p21, PARP (full length and 89 kDa), full length Bid, GPx2 and GAPDH (reference protein) protein expression in HT29 cells after treatment with the medium control (MC), different concentrations of butyrate (2.5-10 mM/40 mM; right panel) and fermentation supernatants (FS) obtained from different dietary fiber samples (FS1: blank control, FS2: Synergy1®, FS3: oat β-glucan, FS4: barley β-glucan, FS5: yeast β-glucan, FS6: Curdlan; left panel) and co-treatment with 5 mM *N*-acetyl-cysteine (NAC). Band sizes are predicted molecular weight band sizes indicated by the suppliers of the antibodies. The real band size may vary moderately.

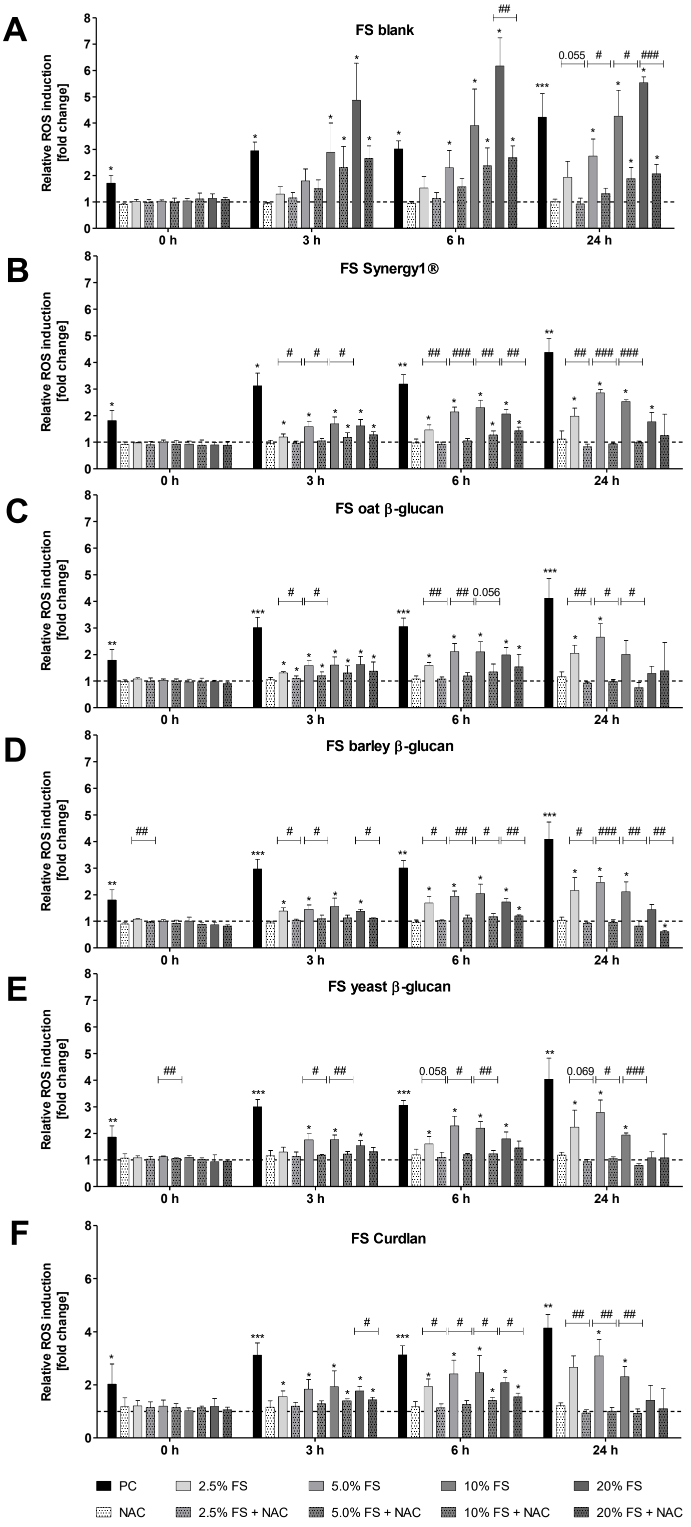

Figure S3. Relative ROS induction (fold change) in LT97 cells after treatment with different concentrations (2.5%, 5%, 10% and 20%) of fermentation supernatants (FS) obtained from the blank control (**A**) and different dietary fiber samples (Synergy1® (**B**), oat β-glucan (**C**), barley β-glucan (**D**), yeast β-glucan (**E**), Curdlan (**F**)) and after co-treatment with 5 mM NAC (*N*-acetyl-cysteine) as well as with the PC (positive control, 2 mM H_2O_2) for 0 h, 3 h, 6 h, 24 h. Relative ROS induction was calculated on the basis of the medium control, which was set 1 (dashed line) (mean + SD, n = 3). Significant differences between cells treated with FS and medium treated cells ($^{*}p < 0.05$) were obtained by Two-Way ANOVA and F-test according to Ryan-Einot-Gabriel-Welsh and significant differences between cells treated with FS alone and cells treated with FS and NAC ($^{\#}p < 0.05$, $^{\#\#}p < 0.01$, $^{\#\#\#}p < 0.001$) were obtained by unpaired Student`s t-test. Significant differences between cells treated with PC and medium treated cells ($^{*}p < 0.05$, $^{**}p < 0.01$, $^{***}p < 0.001$) were obtained by unpaired Student's t-test.

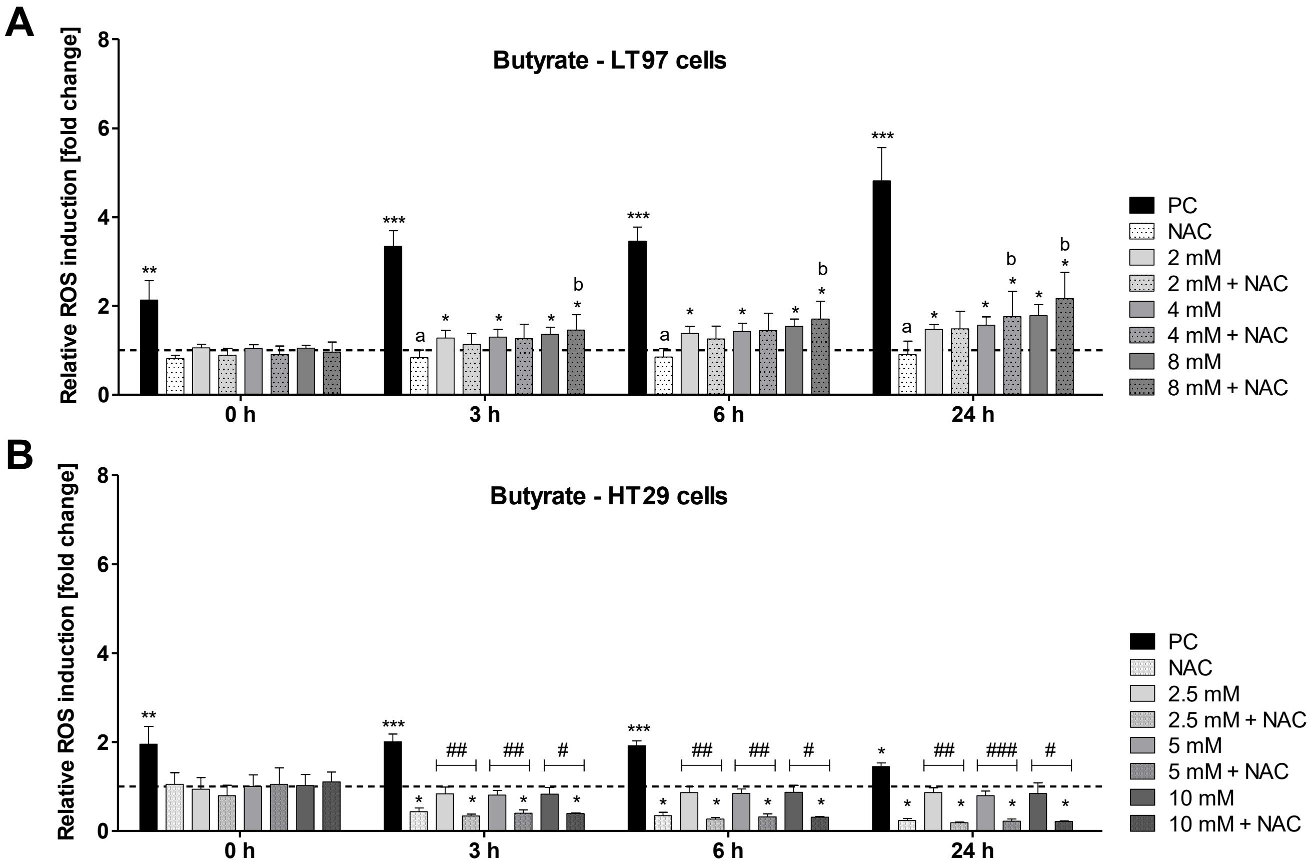

Figure S4. Relative ROS induction (fold change) in LT97 cells after treatment with different concentrations (2 mM, 4 mM, 8 mM) of butyrate and with the PC (positive control, 2 mM H_2O_2) (**A**) and relative ROS induction (fold change) in HT29 cells after treatment with different concentrations (2.5 mM, 5 mM, 10 mM) of butyrate and with the PC (10 mM *tert*-butyl hydroperoxide) (**B**) and co-treatment with 5 mM NAC (*N*-acetyl-cysteine) for 0 h, 3 h, 6 h, 24 h. Relative ROS induction was calculated on the basis of the medium control, which was set 1 (dashed line) (mean + SD, n = 3). Significant differences between cells treated with butyrate and medium treated cells (* $p < 0.05$) and significant differences between cells treated with different concentrations of butyrate or butyrate and NAC (^{a,b} $p < 0.05$, different letters represent significantly different results) were obtained by Two-Way ANOVA and F-test according to Ryan-Einot-Gabriel-Welsh. Significant differences between cells treated with butyrate alone and cells treated with butyrate and NAC ($^{\#}$ $p < 0.05$, $^{\#\#}$ $p < 0.01$, $^{\#\#\#}$ $p < 0.001$) and significant differences between cells treated with the positive controls and medium treated cells (** $p < 0.01$, *** $p < 0.001$) were obtained by unpaired Student's t-test.

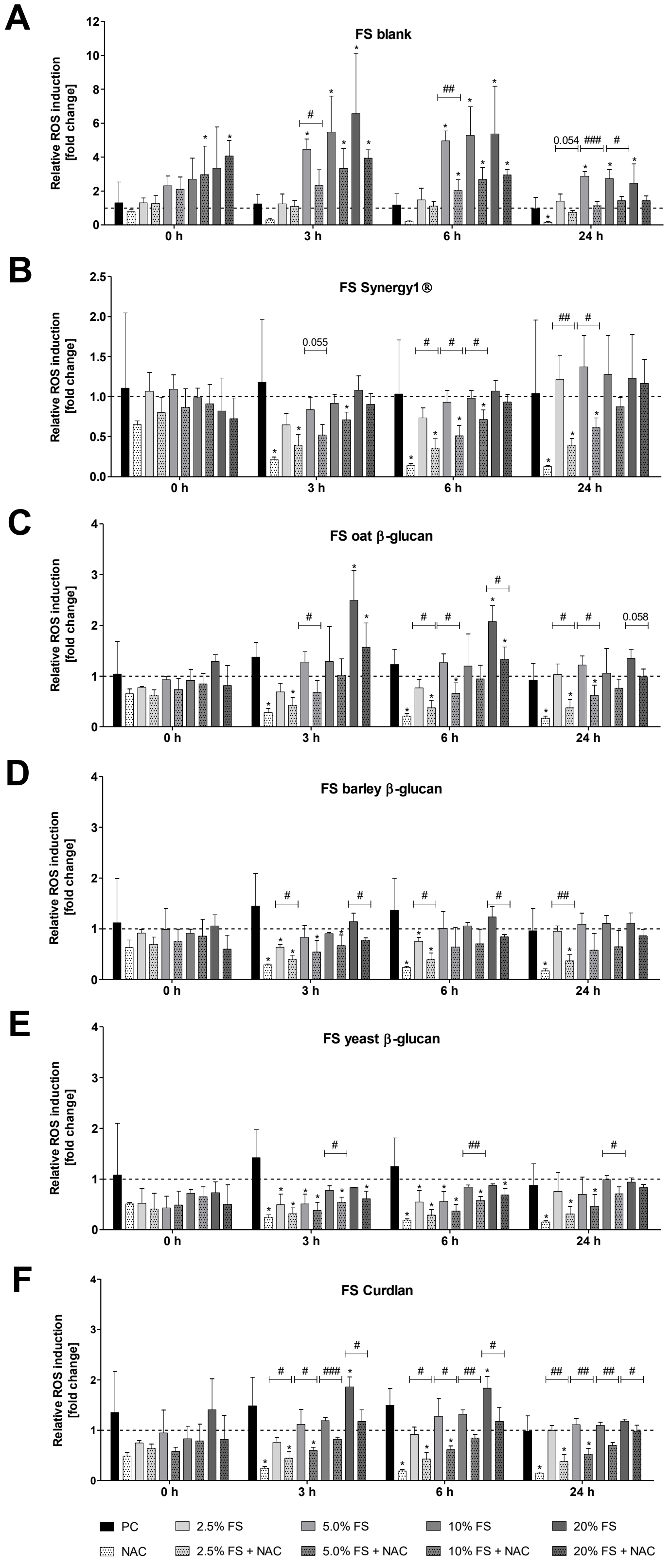

Figure S5. Relative ROS induction (fold change) in HT29 cells after treatment with different concentrations (2.5%, 5%, 10% and 20%) of fermentation supernatants (FS) obtained from the blank control (**A**) and different dietary fiber samples (Synergy1® (**B**), oat β-glucan (**C**), barley β-glucan (**D**), yeast β-glucan (**E**), Curdlan (**F**)) and after co-treatment with 5 mM NAC (*N*-acetyl-cysteine) as well as with the PC (positive control, 10 mM *tert*-butyl hydroperoxide) for 0 h, 3 h, 6 h, 24 h. Relative ROS induction was calculated on the basis of the medium control, which was set 1 (dashed line) (mean + SD, n = 3). Significant differences between cells treated with FS and medium treated cells (* $p < 0.05$) were obtained by Two-Way ANOVA and F-test according to Ryan-Einot-Gabriel-Welsh and significant differences between cells treated with FS alone and cells treated with FS and NAC ($^{\#}$ $p < 0.05$, $^{\#\#}$ $p < 0.01$, $^{\#\#\#}$ $p < 0.001$) were obtained by unpaired Student`s t-test. Significant differences between cells treated with PC and medium treated cells were checked by unpaired Student's t-test.

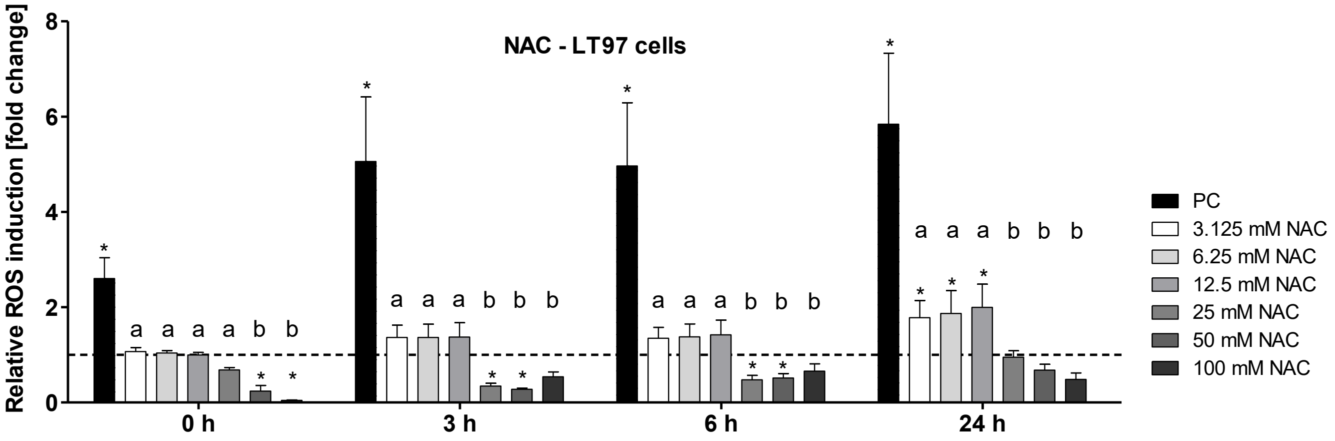

Figure S6. Relative ROS induction (fold change) in LT97 cells after treatment with different concentrations (3.125-100 mM) of NAC (*N*-acetyl-cysteine) and with the PC (positive control, 2 mM H_2O_2) for 0 h, 3 h, 6 h, 24 h. Relative ROS induction was calculated on the basis of the medium control, which was set 1 (dashed line) (mean + SD, n = 3). Significant differences between cells treated with the medium control and cells treated with NAC/PC ($^{*}p < 0.05$) and differences between cells treated with different concentrations of NAC (^{a,b} $p < 0.05$, different letters represent significantly different results) were obtained by One-Way ANOVA and F-test according to Ryan-Einot-Gabriel-Welsh.

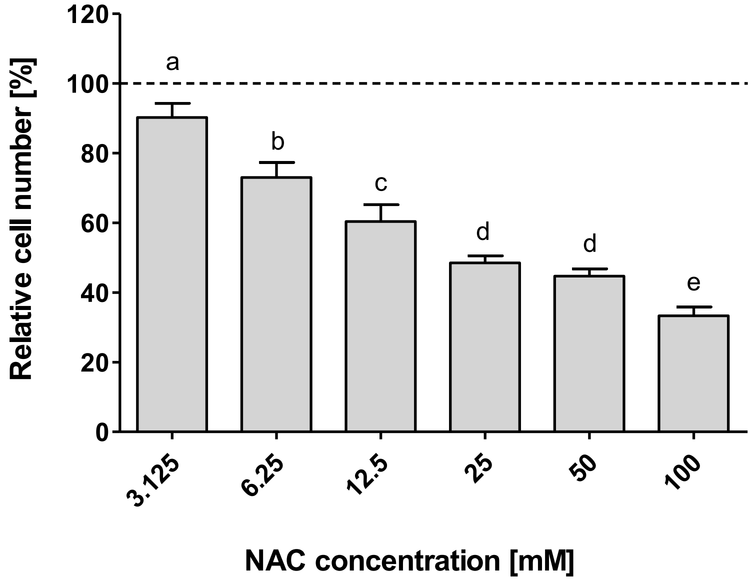

Figure S7. Relative number (%) of LT97 colon adenoma cells after treatment with different concentrations (3.125-100 mM) of NAC (*N*-acetyl-cysteine) for 24 h. Relative cells numbers were calculated on the basis of the medium control (dashed line), which was set 100% (mean + SD, n = 3). Significant differences between cells treated with different concentrations of NAC ($^{a\text{-}e}$ $p < 0.05$, different letters represent significantly different results) were obtained by One-Way ANOVA and F-test according to Ryan-Einot-Gabriel-Welsh.

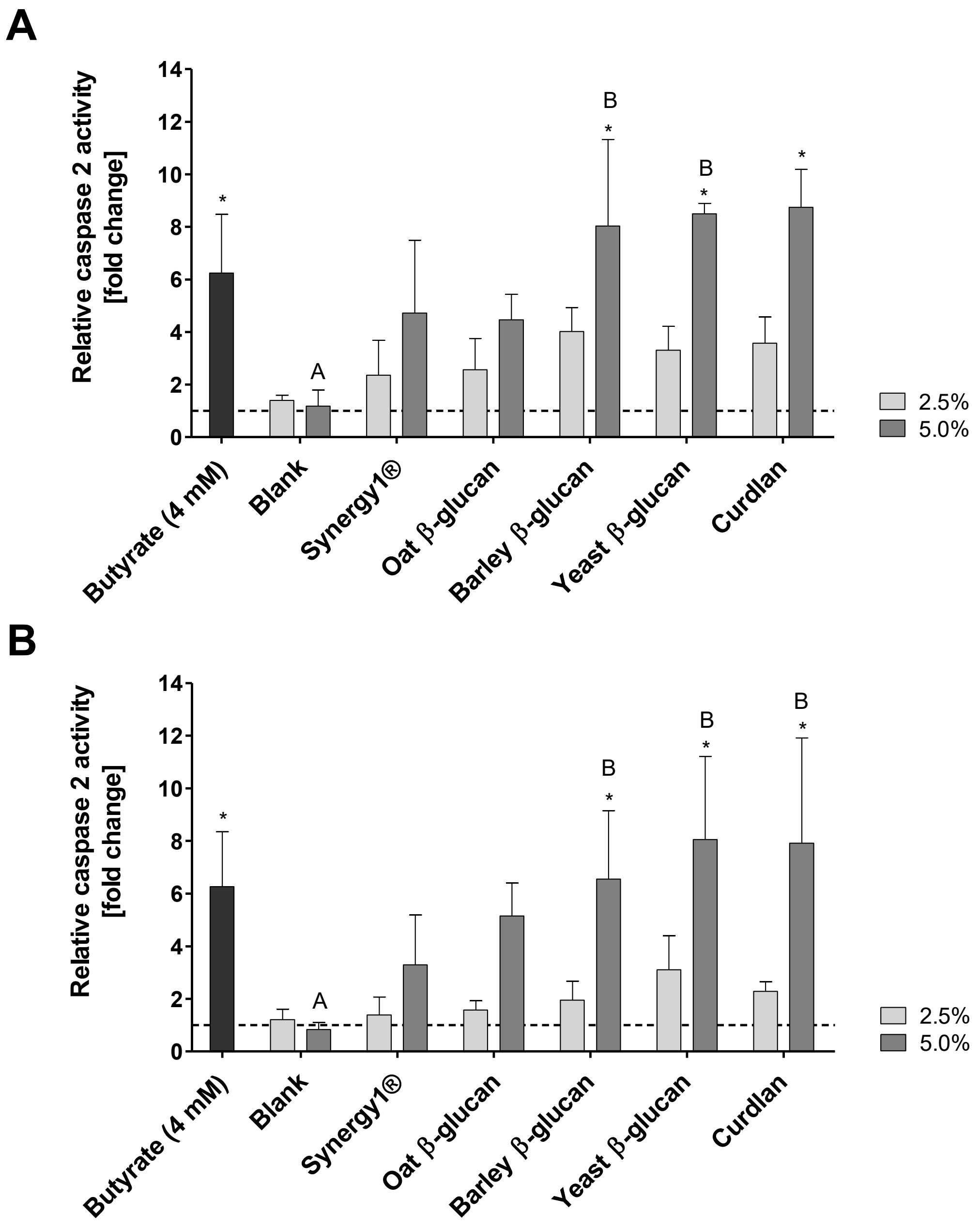

Figure S8. Relative activities of caspase 2 in LT97 cells after treatment with 2.5% and 5% fermentation supernatant (FS) obtained from the blank control and different dietary fiber samples (Synergy1®, oat β-glucan, barley β-glucan, yeast β-glucan, Curdlan) as well as 4 mM butyrate for 24 h (**A**) and 48 h (**B**), (mean + SD, n = 3). Results are presented as fold changes based on a medium control, which was set 1 (dashed line). Significant differences between cells treated with the medium control and cells treated with butyrate and FS ($^{*}p < 0.05$) and differences between cells treated with different FS ($^{A, B}p < 0.05$, different letters represent significantly different results) were obtained by Two-Way ANOVA and F-test according to Ryan-Einot-Gabriel-Welsh.

Table S1. IC_{50}- and IC_{25}-values of LT97 and HT29 cells after treatment with butyrate and FS obtained from different β-glucans.

**Treatment**	**LT97 cells**		**HT29 cells**	
	**IC_{50}**	**IC_{25}**	**IC_{50}**	**IC_{25}**
Butyrate	3.95	0.70	n. d.	3.79
FS blank	9.68	4.00	n. d.	16.61
FS Synergy1®	7.97	1.87	n. d.	11.52
FS oat β-glucan	6.89	1.77	n. d.	8.68
FS barley β-glucan	6.14	1.78	n. d.	10.15
FS yeast β-glucan	6.26	1.44	n. d.	9.16
FS Curdlan	6.80	1.79	n. d.	6.74

IC: inhibitory concentration, FS: fermentation supernatant, n. d.: not detectable
